# Jintiange capsule as add-on therapy for lumbar disc herniation: an open-label randomized controlled trial

**DOI:** 10.3389/fphar.2026.1837527

**Published:** 2026-10-08

**Authors:** Long Liang, Yuxiang Yang, Hao Lv, Yi Wang, Xunlu Yin, Kaiming Li, Xiangwen Gui, Yinghao Zhu

**Affiliations:** 1 The First Affiliated Hospital of Anhui University of Chinese Medicine, Hefei, China; 2 Anhui University of Chinese Medicine, Hefei, China; 3 Wangjing Hospital, China Academy of Chinese Medical Sciences, Beijing, China; 4 Xiyuan Hospital, China Academy of Chinese Medical Sciences, Beijing, China

**Keywords:** jintiange capsules, low back pain, lumbar disc herniation, randomized controlled trial, traditional chinese medicine

## Abstract

**Background:**

Lumbar disc herniation (LDH) is a prevalent and costly disorder. Evidence regarding the efficacy and safety of Jintiange (JTG) capsules as add-on therapy for LDH remains limited.

**Methods:**

This prospective, single-center, open-label randomized add-on trial compared JTG capsules plus conventional therapy with conventional therapy alone. The Japanese Orthopaedic Association (JOA) score was the sole primary outcome; VAS, SF-12 physical and mental component scores (PCS/MCS), numbness, lumbar muscle elastic modulus, and lumbar range of motion (ROM) were secondary outcomes. All 64 randomized participants were included according to their assigned groups. JOA, VAS, PCS, and MCS were analyzed using baseline-adjusted linear mixed-effects models incorporating all available post-baseline observations. Week 8 objective outcomes were analyzed using baseline-adjusted ANCOVA after multiple imputation. Multiplicity across the specified Week 8 secondary outcomes was controlled using the Holm procedure, and complete-case analyses were performed as sensitivity analyses.

**Results:**

Seven participants discontinued follow-up (2 JTG, 5 control). The adjusted between-group difference in JOA favored the JTG-containing regimen at Week 2 (2.50 points, 95% CI 1.03–3.97; P < 0.001), Week 8 (2.73, 95% CI 1.21–4.26; P < 0.001), and 2-week follow-up (1.79, 95% CI 0.27–3.32; P = 0.021). At Week 8, adjusted differences also favored the JTG-containing regimen for VAS (−0.57, 95% CI −0.89 to −0.25), MCS (10.48, 95% CI 9.58–11.39), PCS (4.02, 95% CI 3.34–4.70), lumbar muscle elastic modulus (left −1.22, 95% CI −2.37 to −0.08; right −1.53, 95% CI −2.19 to −0.86), and ROM measures after multiplicity adjustment. Complete-case analyses showed similar effect directions.

**Conclusion:**

The JTG-containing regimen was associated with greater improvement in JOA and several secondary outcomes than conventional therapy alone. However, the open-label, non-placebo-controlled design, undocumented assessor blinding, differential attrition, multiplicity, small sample size, and single-center setting limit causal attribution and generalizability. Further blinded placebo-controlled multicenter trials are warranted.

**Clinical Trial Registration:**

https://www.chictr.org.cn/, ChiCTR2300069735.

## 1 Introduction

Low back pain is a prevalent global health issue, affecting over 80% of the population at some point in their lives (Wu et al., 2020; Wang et al., 2012). A primary cause is lumbar intervertebral disc herniation, a condition that typically progresses with age. Lumbar intervertebral disc herniation accounts for approximately 40% of low back pain cases and is a leading contributor to workforce loss (Jia et al., 2022; GBD, 2017 Disease and Injury Incidence and Prevalence Collaborators, 2018). The associated treatment costs are also considerable, presenting a substantial and often unquantifiable burden for individuals and society. Consequently, preventing low back pain in high-risk populations represents a critical challenge, with the potential to alleviate the significant medical expenses of treatment and rehabilitation (Knezevic et al., 2021).

The pathogenesis of Lumbar disc herniation (LDH) stems from intrinsic factors like aging, reduced cell populations, and extracellular matrix changes, which disrupt intradiscal homeostasis (Vergroesen et al., 2015), as well as from extrinsic factors, including smoking, trauma, obesity, nutritional and metabolic disorders, and abnormal mechanical loading. These extrinsic factors accelerate degeneration by mediating inflammatory responses, apoptosis, pro-inflammatory cytokine secretion, and autophagy (Risbud and Shapiro, 2014; Yang et al., 2020; Ohnishi et al., 2022). A systematic review and meta-analysis identify obesity as a significant risk factor for intervertebral disc herniation, an effect primarily attributed to increased mechanical load on the lumbar spine, though a contribution from metabolic pathways remains possible (Xu et al., 2015; Zhang et al., 2019). Numbness is a common clinical symptom of LDH, with evidence showing that patients with LSS and LBP report more severe hip and leg pain and numbness than those without LBP (Minetama et al., 2022). Currently, most patients with LDH opt for conservative management. While oral analgesics such as non-steroidal anti-inflammatory drugs and muscle relaxants often provide temporary pain relief, the rate of pain recurrence remains high (Carragee et al., 2004).

Traditional Chinese Medicine (TCM) provides distinct strategies for chronic disease management. In TCM theory, disorders associated with LDH are principally ascribed to deficiency syndromes. For example, a deficiency of kidney essence impairs generation and transformation, leading to a failure of the kidneys to govern the bones and resulting in low back and leg pain. Insufficiency of essence and the network vessels, combined with pathogenic stagnation and blood stasis, further manifests as patterns of qi stagnation and blood stasis (Qiu et al., 2017; Xie et al., 2018). Jintiange (JTG) capsules are an approved Chinese medicinal product containing a medicinal ingredient officially designated as “artificial tiger bone powder.” According to an official stamped declaration provided by the manufacturer (Supplementary material 5), the finished product contains no natural tiger bone, and its raw-material components are not derived from tigers (*Panthera tigris*) or tiger tissues. Artificial tiger bone powder is a nationally approved biomimetic pharmaceutical substitute for natural tiger bone and should not be interpreted as tiger-derived medicinal material. JTG capsules have been investigated in musculoskeletal conditions, including osteoporosis (Liang et al., 2022). The formulation primarily comprises inorganic calcium and phosphorus constituents along with organic peptides and proteins (Sun et al., 2019), and JTG is approved in China under National Drug Approval No. Z20030080. The contained bioactive peptides and proteins effectively promote osteoblast differentiation and mineralization (Chen et al., 2022). Previous clinical studies indicate that JTG capsules can treat osteoporosis, rheumatoid arthritis, and osteoarthritis (Yang et al., 2021; Zhuang et al., 2021; Wang et al., 2021). Mechanistic investigations further show that JTG alleviates early-stage glucocorticoid-induced osteonecrosis of the femoral head by preventing subchondral bone destruction, thereby regulating the differentiation of bone marrow mesenchymal stem cells and osteoclast numbers (Xu et al., 2024). However, clinical research on JTG capsules for treating intervertebral disc degeneration is limited, and high-quality experimental evidence supporting its efficacy remains scarce.

We therefore conducted a prospective, single-center, randomized controlled trial to evaluate the efficacy and safety of JTG capsules in patients with intervertebral disc herniation.

## 2 Methods

### 2.1 Trial design

This prospective, single-center, randomized controlled trial was conducted at the Disc Clinic and Spine Department of the First Affiliated Hospital of Anhui University of Chinese Medicine. The study protocol was approved by the hospital’s Medical Ethics Committee (Approval Number:2022AH-49; Supplementary Material 1), and all participants provided written informed consent. The trial was performed in accordance with the principles of the Declaration of Helsinki and Good Clinical Practice guidelines (World Medical Association, 2013). It is registered with the Chinese Clinical Trial Registry (ChiCTR2300069735). To ensure research quality, this trial followed the CONSORT guidelines to guarantee standardized implementation (Butcher et al., 2022). A matching placebo was not used, and participants and treating clinicians were therefore aware of treatment allocation. The original manuscript did not document blinded outcome assessment; assessor blinding was not assumed in this revision.

### 2.2 Participants

Diagnostic Criteria for LDH: Participants must satisfy both imaging and clinical criteria: lumbar MRI or CT must demonstrate a herniated disc with nerve root compression concordant with the symptomatic presentation, in addition to at least three of the following five clinical features: (1) Radiating pain in the lower limb, corresponding to the affected nerve’s distribution; (2) Diminished skin sensation within the corresponding dermatome; (3) A positive straight leg raise test, straight leg raise strengthening test, contralateral straight leg raise test, or femoral nerve traction test; (4) Weakened tendon reflexes relative to the unaffected side; (5) Reduced muscle strength. Furthermore, participants must exhibit symptoms such as low back pain, lower limb pain, numbness, or muscle tension, have a Visual Analogue Scale (VAS) pain score of ≥4, be aged 18–65 years, and provide voluntary written informed consent. Exclusion Criteria: (1) Presence of spondylolisthesis, LDH causing severe cauda equina compression, or clear surgical indications with contraindications to conservative treatment; (2) Other pathologies that could cause low back pain, such as ankylosing spondylitis; (3) Known allergy to the study medications or a general allergic predisposition; (4) Severe cardiovascular, cerebrovascular, hepatic, or renal disease; (5) Psychiatric disorders or conditions such as Alzheimer’s disease that could impair cooperation with the clinical research; (6) Pregnancy or lactation; (7) A history of gastric ulcer or chronic gastritis; (8) Concurrent enrollment in another clinical trial.

### 2.3 Drug and intervention

Jintiange (JTG) Capsules are a commercially manufactured medicinal product approved for clinical use in China (National Drug Approval No. Z20030080). The principal medicinal ingredient is officially designated as “artificial tiger bone powder.” According to an official stamped declaration provided by the manufacturer (Supplementary Material 5), the finished JTG product contains no natural tiger bone, and the raw-material components are not derived from tigers (*P. tigris*) or tiger tissues; therefore, artificial tiger bone powder does not constitute tiger-derived animal medicinal material. Consistent with this declaration, Xu et al. reported that JTG is manufactured from the bones of several farmed edible animal species, including pigs (*Sus scrofa domestica* L.), goats (*Capra hircus* Linnaeus), and deer (*Cervus nippon* Temminck) (Xu et al., 2026). The same study reported that the formulation contains approximately 18% calcium, 18% amino acids, and 8% phosphorus (Xu et al., 2026). Artificial tiger bone powder is a nationally approved biomimetic pharmaceutical substitute developed with reference to selected physicochemical and pharmacological characteristics of natural tiger bone. The capsules used in this study were manufactured by Xi’an Jinhua Pharmaceutical Factory, which holds a GMP certificate and has passed annual audits for ISO9001:2015 and ISO14001:2015. Following the ConPhyMP guidelines, chemical characterization uses pharmacopoeia-compliant methods and orthogonal fingerprinting (Heinrich et al., 2022). Quality-control standards include HPLC identification at 280 nm (no fewer than four major peaks), total amino acids of at least 60 mg per capsule, and calcium content of at least 55 mg per capsule. Ye et al. characterized the amino-acid composition in drug-containing serum using UHPLC-MS/MS (Ye et al., 2025), while Liu et al. identified approximately 400 proteins via HPLC-MS/MS (Liu et al., 2023), including 111 globulins, 53 glycoproteins, 49 binding proteins, 43 membrane proteins, 19 transport proteins, and 13 regulatory proteins. The extraction process involves water extraction of the artificial tiger bone powder (decocted three times with a tenfold volume of water for 2 hours each, followed by spray-drying), yielding a drug-to-extract ratio of approximately 4:1. Each capsule contains 0.4 g of artificial tiger bone powder and the excipient.

In traditional Chinese medicine theory, these capsules are understood to tonify the kidneys, replenish essence, warm and promote circulation to dispel wind, activate blood circulation, unblock collaterals, strengthen tendons, and relieve pain. The treatment group (TG) received Jintiange capsules (three capsules, three times daily) combined with eperisone (one tablet, three times daily) for eight consecutive weeks, with celecoxib (one tablet, twice daily) administered for the initial 2 weeks. The control group (CG) was treated with eperisone (one tablet, three times daily) for eight consecutive weeks, along with celecoxib (one tablet, twice daily) for the first 2 weeks.

The 2-week course of celecoxib was predetermined in the study protocol. This duration was chosen based on the drug label indication for acute pain (generally not exceeding 2 weeks)and consistent with clinical practice guidelines recommending short-term use of NSAIDs for acute low back pain. After the 2-week celecoxib course, both groups discontinued celecoxib and continued only on their respective maintenance regimens (JTG + eperisone in the treatment group; eperisone alone in the control group). Importantly, the celecoxib regimen was identical between the two groups in terms of dosage, frequency, and duration, ensuring that any between-group differences in outcomes after week 2 could not be attributed to differential analgesic use. The protocol allowed for resumption of celecoxib if clinically indicated; however, no patient in either group required additional celecoxib during the trial.

All Jintiange Capsules administered to participants in the treatment group were obtained from a single manufacturing batch (batch No.260203) produced by Xi’an Jinhua Pharmaceutical Factory. The batch was released in accordance with the national drug standard YBZ01592003-2009Z. The corresponding batch release documentation confirmed compliance with the specified quality-control requirements, including HPLC identification at 280 nm (no fewer than four major peaks), total amino acids ≥60 mg per capsule, calcium content ≥55 mg per capsule, and the applicable specifications for moisture and heavy metals (Supplementary material 4).

Regarding upstream sourcing and traceability, the manufacturer’s official declaration states that the company maintains an upstream supplier-management system and retains relevant quality documentation, including raw-material procurement contracts, inspection reports, incoming and outgoing inventory records, and supply-chain traceability records. These documents are available for inspection by the competent drug regulatory authorities. Detailed supplier identities and commercially sensitive procurement information constitute proprietary commercial information and are therefore not publicly disclosed. The investigators did not procure, breed, sacrifice, or process any animals for the purpose of this clinical trial; participants received only the finished, commercially manufactured JTG pharmaceutical product. The original stamped manufacturer declaration is provided as Supplementary Material 5.

Potential drug-drug interactions: No dedicated clinical pharmacokinetic interaction study has specifically evaluated the coadministration of JTG with celecoxib or eperisone. Celecoxib is metabolized predominantly by CYP2C9 and may inhibit CYP2D6 (Zhang et al., 2026), whereas eperisone undergoes extensive first-pass metabolism involving multiple CYP enzymes, including CYP2J2 and CYP3A4 (Yoo et al., 2009). JTG is a complex pharmaceutical preparation containing mineral and protein/peptide constituents; however, its human CYP-mediated interaction profile has not been established. Accordingly, clinically relevant pharmacokinetic interactions cannot be formally excluded. Pharmacodynamically, the analgesic and anti-inflammatory actions reported for JTG may add to the analgesic/anti-inflammatory effect of celecoxib, while its musculoskeletal effects may overlap with those of eperisone. Therefore, the clinical effects observed in this study are interpreted as effects of the combination regimen rather than as the effects of JTG monotherapy.

It should be noted that due to the distinctive organoleptic properties (odor, taste, and appearance) of JTG capsules—attributable to their natural animal-origin components—and ethical considerations regarding the use of an approved drug in symptomatic patients, a double-blind, double-dummy placebo-controlled design was not implemented in this trial. Consequently, the control group did not receive placebo capsules matching the JTG intervention, resulting in unequal pill burden between groups.

### 2.4 Randomization

A professional statistician generated 64 random number sequences using a random number table. These sequences were assigned sequentially according to each participant’s order of enrollment, randomizing the 64 patients with LDH in a 1:1 ratio to either the experimental group (Jintiange capsules plus celecoxib and eperisone) or the CG (celecoxib and eperisone), with 32 patients per group. To ensure allocation concealment, the random codes and corresponding group assignments were sealed within sequentially numbered, opaque envelopes.

### 2.5 Sample size calculation

The sample size was determined using the JOA score as the primary endpoint. A two-group mean comparison formula was applied, with α set to 0.05 (two-tailed Zα = 1.96) and β to 0.1 (power = 90%, Zβ = 1.28). Based on prior literature, the standard deviation (σ) of the JOA score in the CG was 2.84. The between-group difference (δ) was defined as 2.5, corresponding to the reported minimal clinically important difference (MCID) for the JOA score (Kim et al., 2019). This calculation yielded a requirement of 27 participants per group, or 54 participants in total. To accommodate an estimated 20% dropout rate, the final target sample size was set at approximately 64 participants.

### 2.6 Outcome measures

The primary outcome measure is the Japanese Orthopaedic Association (JOA) Score, a core standardized tool for assessing symptoms, function, and treatment efficacy in patients with lumbar spine conditions. Secondary outcome measures comprise the Visual Analogue Scale (VAS) for low back pain intensity, the Likert-type numbness severity Scale for assessing numbness in the low back and lower limbs, and the Short Form Health Survey (SF-12), which evaluates overall quality of life through its Physical (PCS) and Mental (MCS) Component Summaries. Additional measures include ultrasound-based assessment of the lumbar muscle elastic modulus and lumbar range of motion—specifically forward flexion, backward extension, and left and right lateral flexion—measured with a lumbar motion device. We assessed the JOA, VAS, Richter, and SF-12 scores at baseline, during treatment at weeks 2 and 8, and at a follow-up 2 weeks after treatment concluded. The lumbar muscle elastic modulus and range of motion were measured solely at baseline and after the 8-week treatment period. For the present re-analysis, Week 8 was selected as the principal post-treatment contrast for secondary outcomes to facilitate multiplicity control.

### 2.7 Safety indicators

Before trial initiation, we prospectively assessed the safety of Jintiange Capsules based on the national drug standard (YBZ01592003-2009Z), package insert, and published clinical studies. According to the insert (Supplementary Material 2), the most common adverse reactions are dry mouth, constipation, and mild gastrointestinal discomfort; No well-established drug-drug interactions between JTG and celecoxib or eperisone have been documented. Nevertheless, dedicated clinical pharmacokinetic interaction studies investigating this combination are unavailable, and the product is contraindicated for those allergic to any ingredient. The manufacturer holds a valid GMP certificate and ISO9001:2015 certification. Each batch must pass all quality control tests (HPLC identification, total amino acids, calcium content, moisture, and heavy metals) before release. Because the same manufacturing batch was used for all participants receiving JTG, potential variability arising from between-batch differences was minimized. Based on this assessment, the protocol received ethics committee approval, with prespecified safety monitoring parameters including liver function (ALT, AST), renal function (Cr, BUN), and routine blood tests at baseline and week 8.

Safety indicators included the recording of all adverse events. We specifically focused on monitoring serious adverse events, primarily including loss of participant function, life-threatening events, and drug-induced nausea and vomiting reactions. All adverse events were classified according to their severity (mild, moderate, severe) based on general clinical standards. In the event of a death, an independent physician was evaluated to analyze its potential causal relationship and association with the study intervention, and a written report was submitted to the local drug regulatory authority.

### 2.8 Statistical analysis

Statistical modeling was performed using the analysis dataset in Python (statsmodels 0.14.6 and SciPy). Continuous variables were summarized as mean ± standard deviation (SD), ordinal variables as median [interquartile range, IQR], and categorical variables as counts and percentages. Baseline comparisons were descriptive and were not used to determine inclusion in the efficacy models.

The primary analysis followed an intention-to-treat (ITT) principle, including all 64 analytic records (32 per group). For the JOA, VAS, PCS, and MCS outcomes, post-baseline measurements at Week 2, Week 8, and follow-up were analyzed using baseline-adjusted linear mixed-effects models with a subject-specific random intercept. Fixed effects included treatment group, visit, the treatment-by-visit interaction, and the corresponding baseline value. Models were estimated using restricted maximum likelihood (REML). This likelihood-based approach incorporated all available observed values and did not use single-value imputation, such as last observation carried forward, to replace missing Week 8 or follow-up measurements.

For each post-baseline visit, adjusted marginal means, adjusted between-group differences, 95% confidence intervals, and two-sided P values were estimated. In addition, a separate complete-case sensitivity analysis was conducted using the 57 participants with complete follow-up data, including 30 participants in the treatment group and 27 in the control group. Consistency in the direction and magnitude of the estimated treatment effects between the ITT-oriented and complete-case analyses was evaluated as a measure of robustness.

Lumbar muscle elastic modulus and lumbar range of motion (ROM) were measured only at baseline and Week 8. Because Week 8 measurements were missing for some records, these outcomes were analyzed within the ITT-oriented sensitivity-analysis framework using 200 multiple imputations. The imputation models included treatment group, the baseline value of the corresponding outcome, and baseline and Week 2 values of JOA, VAS, PCS, and MCS. Each imputed dataset was analyzed using an analysis of covariance (ANCOVA) model for the Week 8 outcome adjusted for its corresponding baseline value, and the estimates were subsequently pooled according to Rubin’s rules.

The Likert-type numbness score was treated as an ordinal outcome. Between-group comparisons at each visit were performed using the Mann–Whitney U test, with Bonferroni correction applied to the three post-baseline comparisons. Within-group changes across the four time points were assessed using the Friedman test among complete cases. For the family of secondary outcomes assessed at Week 8, including VAS, MCS, PCS, numbness score, two lumbar muscle elastic-modulus measures, and four lumbar ROM measures, a total of 10 statistical tests were adjusted using the Holm procedure. Two-sided adjusted P values < 0.05 were considered statistically significant.

## 3 Results

### 3.1 Participants and baseline characteristics

This trial initially screened 70 patients for eligibility; six were excluded and 64 were randomized in a 1:1 ratio (32 per group). Seven participants discontinued during follow-up (2 in the treatment group and 5 in the control group). All 64 randomized participants were retained in the ITT population and analyzed according to their original treatment allocation. Measurements obtained before withdrawal were retained, whereas measurements not obtained after withdrawal were treated as missing. Accordingly, the ITT-oriented analytic dataset contained 64 records, whereas the complete-case sensitivity dataset contained 57 records (30 treatment and 27 control). The participant recruitment, allocation, follow-up, and analysis process is shown in Figure 1. The numerically greater number of withdrawals in the control group was retained as a potential source of attrition bias rather than being interpreted as resolved by the statistical model The baseline demographic and clinical characteristics of the participants are summarized in Table 1.

**FIGURE 1:**
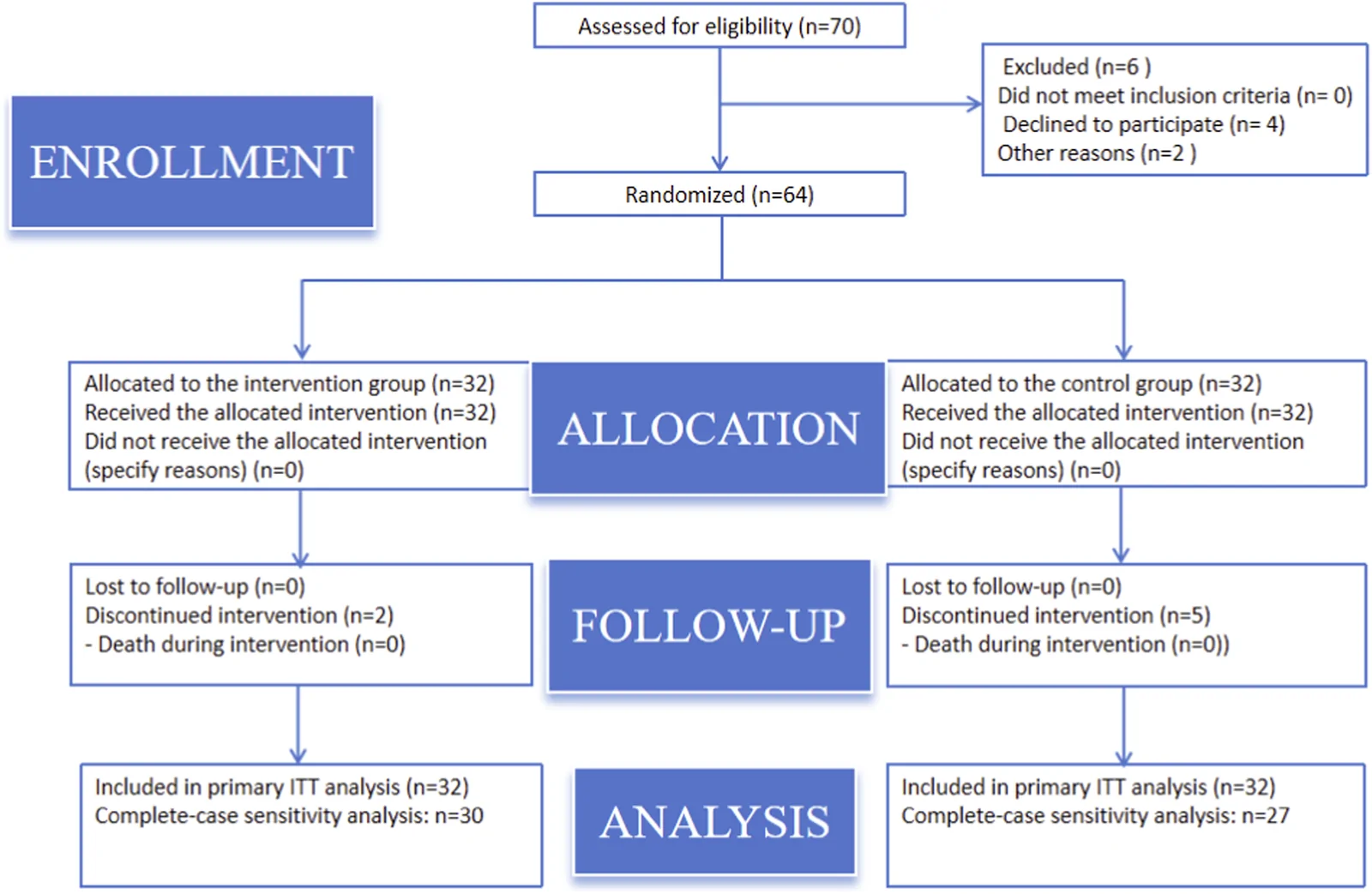
Patient recruitment and follow-up flowchart.

**TABLE 1:** Demographic baseline characteristics of patients.

Items	Treatment (n = 32)	Control (n = 32)	*Descriptive P*
Gender (male/female)	15/17	13/19	0.801
Age (years)	48.59 ± 16.33	44.78 ± 13.73	0.316
Disease duration (years), median [IQR]	3.00 [2.38, 5.00]	3.00 [2.00, 4.25]	0.886
JOA	8.91 ± 3.34	8.66 ± 3.43	0.769
VAS	6.84 ± 0.83	7.05 ± 0.61	0.260
SF-12 PCS	21.81 ± 4.94	21.59 ± 3.68	0.842
SF-12 MCS	22.59 ± 5.09	22.06 ± 3.82	0.638
Likert numbness, median [IQR]	3 [2, 3]	2 [2, 3]	0.138
Elastic modulus, left	19.31 ± 3.20	19.07 ± 2.35	0.733
Elastic modulus, right	19.73 ± 2.14	19.43 ± 1.95	0.553
ROM: forward flexion	32.50 ± 3.54	32.64 ± 6.98	0.923
ROM: backward extension	19.87 ± 2.74	19.86 ± 3.33	0.986
ROM: left lateral flexion	18.29 ± 2.84	17.28 ± 3.29	0.196
ROM: right lateral flexion	18.05 ± 2.96	16.90 ± 3.12	0.134

P values for continuous baseline variables were calculated using Welch’s t-test; disease duration and Likert-type numbness scores were analyzed using the Mann–Whitney U test; and sex was analyzed using Fisher’s exact test. Baseline P values are provided for descriptive purposes only.

Continuous outcomes were analyzed on their observed numeric scales using the prespecified parametric models. Model assumptions were assessed through inspection of residual distributions, histograms, and Q–Q plots, and no substantial departures from normality were identified for JOA, VAS, SF-12 (PCS and MCS), elastic modulus, or lumbar range of motion. The Likert-type numbness score was treated as an ordinal outcome and was therefore analyzed using non-parametric methods, irrespective of formal normality testing. Detailed distributional assessments are provided in Supplementary Table S3. The inferential treatment-effect estimates reported below were derived from the specified analysis models rather than from baseline significance testing.

Regarding post-protocol analgesic use, no patient in either group required rescue analgesia with celecoxib or other NSAIDs after the initial 2-week treatment period. Thus, the between-group comparisons of outcomes at weeks 2, 8, and the 2-week follow-up were not confounded by differential use of additional analgesics.

### 3.2 Primary outcome

The primary outcome was the JOA score (Table 2). In the ITT baseline-adjusted linear mixed-effects analysis including all available observations from the 64 randomized participants, the adjusted between-group difference favored the JTG-containing regimen at week 2 (2.50 points, 95% CI 1.03–3.97; P < 0.001), week 8 (2.73 points, 95% CI 1.21–4.26; P < 0.001), and the 2-week post-treatment follow-up (1.79 points, 95% CI 0.27–3.32; P = 0.021) (Figure 2A). The treatment-by-visit interaction across the three post-baseline visits was not statistically significant (Wald χ^2^ = 2.09, df = 2, P = 0.353), indicating that there was no statistically significant evidence that the magnitude of the between-group treatment effect differed across post-baseline visits. The complete-case sensitivity analysis showed treatment-effect estimates in the same direction. At week 8, the adjusted between-group difference in the complete-case analysis was 2.91 points (95% CI 1.36–4.46; P < 0.001), compared with 2.73 points in the ITT-oriented analysis.

**TABLE 2:** Primary JOA outcome: intention-to-treat baseline-adjusted linear mixed-effects analysis.

Visit	Treatment adjusted mean	Control adjusted mean	Adjusted difference	95% CI	P
Week 2	16.91	14.40	2.50	1.03 to 3.97	<0.001
Week 8	23.32	20.59	2.73	1.21 to 4.26	<0.001
2-week follow-up	24.46	22.66	1.79	0.27 to 3.32	0.021

Adjusted marginal means were estimated from the baseline-adjusted linear mixed-effects model under the intention-to-treat principle. All 64 randomized participants were retained in the ITT, population according to their original treatment allocation. All available post-baseline observations were included. Missing week 8 and follow-up measurements were not replaced using LOCF, or other single-value imputation methods and were handled through likelihood-based mixed-effects modeling. The complete-case analysis was performed as a sensitivity analysis.

**FIGURE 2:**
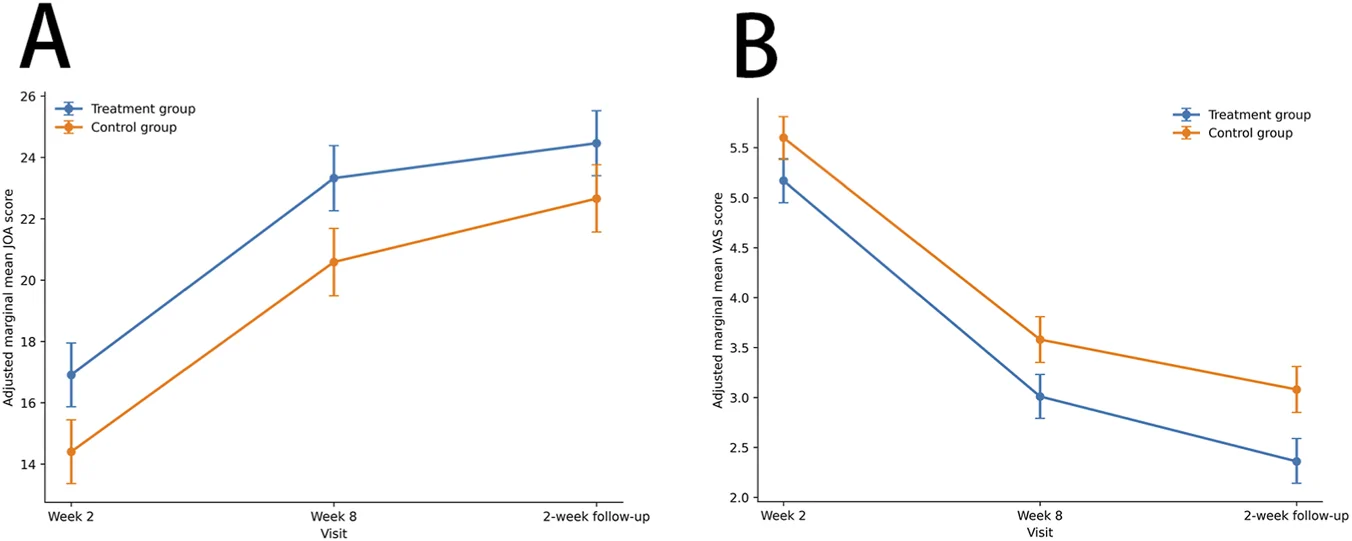
Adjusted longitudinal trajectories of JOA and VAS scores. **(A)** Adjusted marginal mean Japanese Orthopaedic Association (JOA) scores at week 2, week 8, and the 2-week post-treatment follow-up. **(B)** Adjusted marginal mean visual analogue scale (VAS) scores at week 2, week 8, and the 2-week post-treatment follow-up. Points represent adjusted marginal means estimated from baseline-adjusted linear mixed-effects models, and error bars represent 95% confidence intervals. Treatment-group differences were estimated within the same mixed-effects modeling framework. Higher JOA scores and lower VAS scores indicate better clinical outcomes.

### 3.3 Secondary outcomes

Secondary continuous longitudinal outcomes were analyzed according to the same ITT mixed-effects framework (Table 3).

1. VAS favored the JTG-containing regimen at week 2 (adjusted difference −0.43, 95% CI −0.73 to −0.12; P = 0.006), week 8 (−0.57, 95% CI −0.89 to −0.25; P < 0.001), and follow-up (−0.72, 95% CI −1.04 to −0.40; P < 0.001) (Figure 2B). The treatment-by-visit interaction was not significant for VAS (P = 0.180), indicating a relatively stable between-group difference across post-baseline visits.
2. For SF-12 MCS, the adjusted treatment differences were 8.36 points at Week 2 (95% CI 7.46–9.25), 10.48 at week 8 (95% CI 9.58–11.39), and 8.98 at follow-up (95% CI 8.08–9.89), all P < 0.001. The treatment-by-visit interaction was significant (P < 0.001), reflecting variation in the magnitude of the group difference across visits.
3. For SF-12 PCS, the adjusted treatment differences were 11.63 points at Week 2 (95% CI 10.96–12.29), 4.02 at week 8 (95% CI 3.34–4.70), and 3.62 at follow-up (95% CI 2.94–4.30), all P < 0.001. The treatment-by-visit interaction was significant (P < 0.001) (Figure 3A). Complete-case sensitivity models for VAS, MCS, and PCS generated effects in the same direction and of similar magnitude.

**TABLE 3:** Secondary outcomes in the intention-to-treat and sensitivity analyses.

Outcome/visit	Analysis	Adjusted effect (TG−CG)	95% CI	Raw P	Holm P*
VAS – week 2	LMM	−0.43	−0.73 to −0.12	0.006	—
VAS – week 8	LMM	−0.57	−0.89 to −0.25	<0.001	0.003
VAS – follow-up	LMM	−0.72	−1.04 to −0.40	<0.001	—
MCS – week 2	LMM	8.36	7.46 to 9.25	<0.001	—
MCS – week 8	LMM	10.48	9.58 to 11.39	<0.001	<0.001
MCS – follow-up	LMM	8.98	8.08 to 9.89	<0.001	—
PCS – week 2	LMM	11.63	10.96 to 12.29	<0.001	—
PCS – week 8	LMM	4.02	3.34 to 4.70	<0.001	<0.001
PCS – follow-up	LMM	3.62	2.94 to 4.30	<0.001	—
Elastic modulus – left, week 8	MI + ANCOVA	−1.22	−2.37 to −0.08	0.036	0.036
Elastic modulus – right, week 8	MI + ANCOVA	−1.53	−2.19 to −0.86	<0.001	<0.001
ROM forward flexion – week 8	MI + ANCOVA	5.87	2.13 to 9.60	0.002	0.011
ROM backward extension – week 8	MI + ANCOVA	2.27	0.41 to 4.14	0.017	0.034
ROM left lateral flexion – week 8	MI + ANCOVA	1.16	0.32 to 2.00	0.007	0.028
ROM right lateral flexion – week 8	MI + ANCOVA	1.44	0.35 to 2.52	0.009	0.028

Holm-adjusted P values are reported only for the family of 10 secondary outcomes assessed at week 8, including VAS, MCS, PCS, the Likert-type numbness score (reported in Table 4 below), two lumbar muscle elastic-modulus measures, and four lumbar range of motion (ROM) measures. Comparisons at week 2 and follow-up were considered supportive longitudinal contrasts and were therefore not included in this week 8 multiplicity-adjustment family.

**TABLE 4:** Likert-type numbness score: available-case Mann–Whitney U comparisons.

Visit	Treatment	Control	N (TG/CG)	U	Raw P	Bonferroni P
Baseline	3 [2, 3]	2 [2, 3]	32/32	608	0.138	—
Week 2	2 [1, 2]	2 [2, 2]	32/32	256.5	<0.001	<0.001
Week 8	0.5 [0, 1]	1 [1, 1]	30/27	165	<0.001	<0.001
2-week follow-up	1 [1, 2]	2 [1, 2]	30/27	317	0.110	0.329

Values are median [IQR]. Baseline is descriptive. Post-baseline Mann–Whitney U P values were Bonferroni-corrected across week 2, week 8, and follow-up. Friedman tests in the 57 complete cases showed significant within-group changes over time in both the treatment group (χ^2^ = 78.45, P < 0.001) and control group (χ^2^ = 67.58, P < 0.001).

**FIGURE 3:**
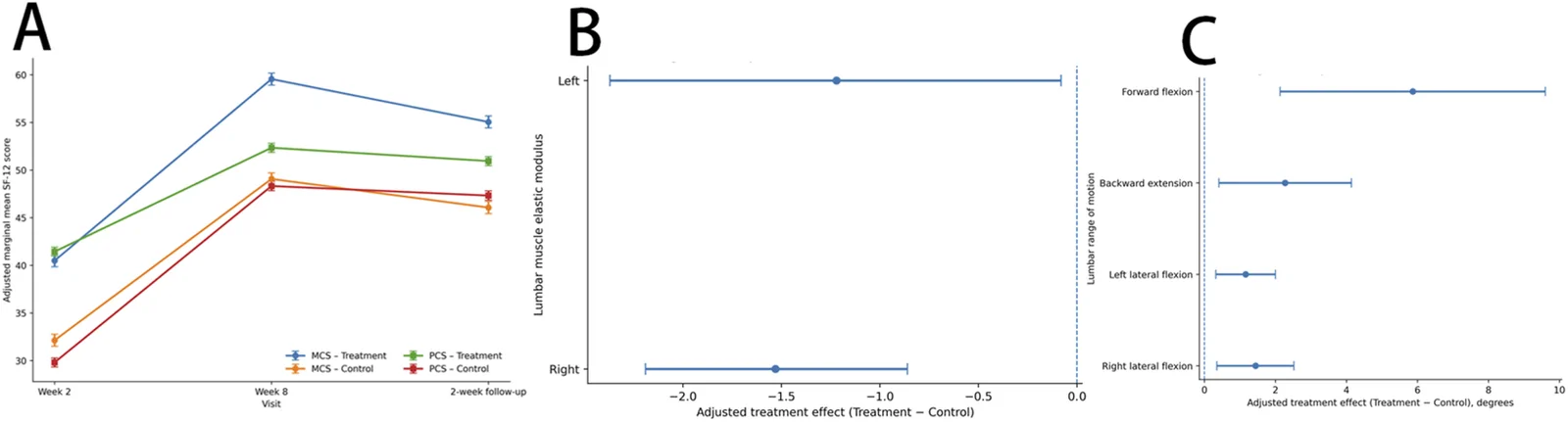
Adjusted secondary outcomes during treatment and follow-up. **(A)** Adjusted marginal mean SF-12 Mental Component Summary (MCS) and Physical Component Summary (PCS) scores at week 2, week 8, and the 2-week post-treatment follow-up. Estimates were obtained from baseline-adjusted linear mixed-effects models using all available post-baseline observations. **(B)** Adjusted between-group differences in left and right lumbar muscle elastic modulus at week 8, estimated using multiple imputation followed by baseline-adjusted analysis of covariance (ANCOVA). **(C)** Adjusted between-group differences in lumbar range of motion (ROM), including forward flexion, backward extension, left lateral flexion, and right lateral flexion, at week 8, estimated using multiple imputation followed by baseline-adjusted ANCOVA. In **(A)**, points represent adjusted marginal means and error bars represent 95% confidence intervals (CIs). In **(B,C)**, points represent adjusted treatment effects calculated as treatment minus control, and horizontal error bars represent 95% CIs. The vertical dashed line at zero indicates no between-group difference. For elastic modulus, negative values favor the treatment group, whereas for ROM, positive values favor the treatment group. SF-12, 12-Item Short Form Health Survey; MCS, Mental Component Summary; PCS, Physical Component Summary; ROM, range of motion.

LMM refers to a baseline-adjusted linear mixed-effects model incorporating all available post-baseline observations. MI + ANCOVA refers to 200-fold multiple imputation followed by baseline-adjusted analysis of covariance (ANCOVA) for the week 8 outcome, with estimates pooled according to Rubin’s rules.

For the multiple-imputation analyses, participants who discontinued follow-up and therefore lacked week 8 objective-outcome measurements retained all observed baseline and week 2 information. Missing week 8 objective outcomes were multiply imputed using treatment allocation, the corresponding baseline outcome value, and available baseline and week 2 values of JOA, VAS, MCS, and PCS as predictors. Estimates from 200 imputed datasets were pooled according to Rubin’s rules.

All effect estimates were calculated as treatment minus control. Therefore, negative differences in lumbar muscle elastic modulus and VAS favored the treatment group, whereas positive differences in JOA, MCS, PCS, and ROM favored the treatment group.

Because the objective outcomes were assessed only at baseline and week 8, their ITT analyses were based on multiple imputation rather than the repeated-measures linear mixed-effects models used for JOA, VAS, MCS, and PCS.

4. At Week 8, the multiply imputed baseline-adjusted analysis estimated lower lumbar muscle elastic modulus in the treatment group than in the control group (left: adjusted difference −1.22, 95% CI −2.37 to −0.08, Holm-adjusted P = 0.036; right: −1.53, 95% CI −2.19 to −0.86, Holm-adjusted P < 0.001) (Figure 3B). Lumbar ROM favored the treatment group for forward flexion (5.87, 95% CI 2.13 to 9.60; Holm P = 0.011), backward extension (2.27, 95% CI 0.41 to 4.14; Holm P = 0.034), left lateral flexion (1.16, 95% CI 0.32 to 2.00; Holm P = 0.028), and right lateral flexion (1.44, 95% CI 0.35 to 2.52; Holm P = 0.028) (Figure 3C).

The Likert-type numbness score was analyzed as an ordinal secondary outcome. The method-validation withdrawal records contributed their available baseline and week 2 values only; week 8 and follow-up comparisons therefore used 30 treatment and 27 control complete observations.

5. Numbness scores favored the treatment group during treatment. At Week 2, the treatment median was 2 (Wu et al., 2020; Wang et al., 2012) versus 2 (Wang et al., 2012Wang et al., 2012) in controls (Bonferroni-adjusted P < 0.001), and at week 8 the medians were 0.5 (0, 1) versus 1 (Wu et al., 2020Wu et al., 2020) (adjusted P < 0.001). At the 2-week post-treatment follow-up, the between-group difference was no longer statistically significant (1 (Wu et al., 2020; Wang et al., 2012) vs. 2 (Wu et al., 2020; Wang et al., 2012); adjusted P = 0.329). The week 8 numbness result also remained significant within the 10-outcome Holm-adjusted secondary family (Holm P < 0.001) (Figure 4).

**FIGURE 4:**
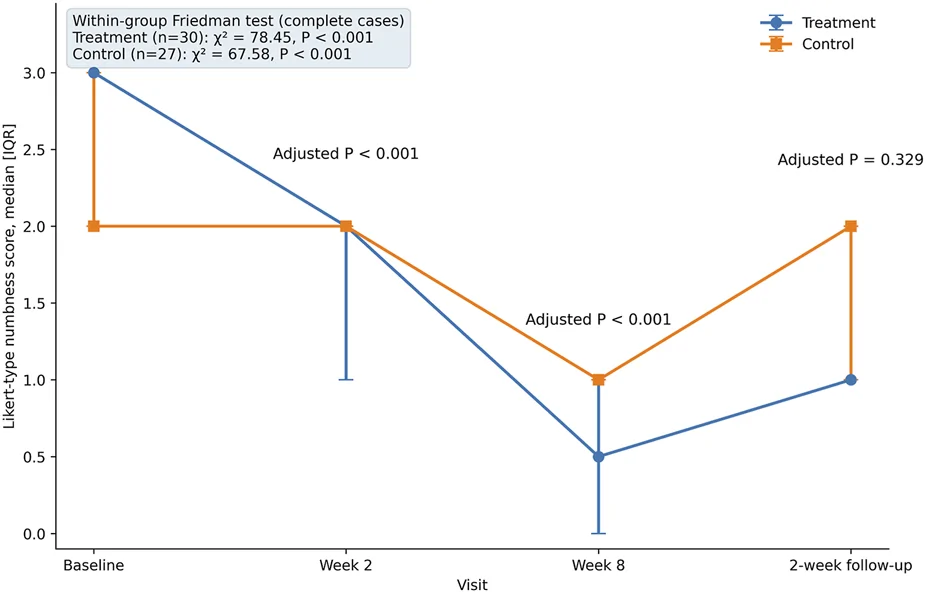
Changes in numbness scores during treatment and follow-up. Numbness was assessed using a Likert-type ordinal scale at baseline, week 2, week 8, and the 2-week post-treatment follow-up. Data are presented as medians with interquartile ranges (IQRs). Changes over time within each group were evaluated using the Friedman test, which demonstrated significant overall time effects in both the treatment group (χ^2^ = 78.45, P < 0.001) and the control group (χ^2^ = 67.58, P < 0.001). Between-group comparisons at post-baseline visits were performed using the Mann–Whitney U test with Bonferroni correction. Significant between-group differences were observed at week 2 and week 8 (both adjusted P < 0.001), whereas no significant between-group difference was observed at the 2-week follow-up (adjusted P = 0.329). Error bars represent the interquartile range.

### 3.4 Safety outcomes

No serious adverse events were reported in the observed safety dataset. Safety assessments included vital signs, routine blood tests, liver function, and renal function at baseline and week 8. Mild gastrointestinal discomfort occurred in two participants in the treatment group and one participant in the control group, and all events resolved spontaneously. Because seven participants discontinued follow-up, subsequent adverse-event and laboratory data were unavailable for these participants. Missing safety data were therefore neither artificially assigned nor imputed, and these missing observations were not included in inferential safety analyses. Safety outcomes were summarized descriptively based only on actually observed data and were not incorporated into the ITT-oriented efficacy analysis framework.

No clinically apparent safety signal suggestive of a drug-drug interaction was identified during the study. However, because plasma concentrations of JTG constituents, celecoxib, and eperisone were not measured, these clinical safety observations cannot exclude a pharmacokinetic interaction.

## 4 Discussion

In the present re-analysis, the principal statistical change was the adoption of an intention-to-treat framework in which all 64 randomized participants were retained according to their original treatment allocation and all available post-randomization observations were used. Baseline-adjusted mixed-effects modeling was applied to the longitudinal JOA, VAS, PCS, and MCS outcomes, thereby allowing participants with incomplete follow-up to contribute their available measurements without requiring LOCF. The complete-case analysis was retained as a sensitivity analysis to evaluate the robustness of the primary findings.

The pathological mechanism of LDH involves degenerative changes in the nucleus pulposus, ruptures of the annulus fibrosus and cartilaginous endplates, and a consequent imbalance in the disc’s internal stress distribution. This process leads to the release of inflammatory mediators, which stimulate nerve endings in the outer annulus fibrosus and nociceptors beneath the endplates (Wiet et al., 2017; Hazer et al., 2021). Conservative management remains the primary treatment for LDH, with first-line pharmacological therapy typically employing non-steroidal anti-inflammatory drugs (NSAIDs) and muscle relaxants to control low back and radicular pain from nerve root inflammation. However, the long-term use of NSAIDs is constrained by gastrointestinal toxicity, such as bleeding and ulcers, and is linked to a high recurrence rate (Bydon et al., 2014; Ko and Kim, 2025). Traction therapy is another common treatment due to its low cost, operational simplicity, safety, and high patient acceptance. Research by Schimmel et al. confirms that lumbar traction can improve functional mobility and alleviate pain in LDH patients (Mitchell et al., 2021; Schimmel et al., 2009). Celecoxib, a selective COX-2 inhibitor, provides anti-inflammatory, analgesic, and antipyretic effects and is FDA-approved for acute pain conditions like primary dysmenorrhea (Wang et al., 2022). Its oral formulation alleviates joint pain and reduces local tissue inflammation by inhibiting inflammatory mediator synthesis (Krasselt and Baerwald, 2019). Furthermore, a systematic review and meta-analysis by Charlotte Beaudart et al., using databases including MEDLINE, Cochrane Central, and Scopus, concluded that celecoxib demonstrates superior safety and efficacy versus non-selective NSAIDs in treating osteoarthritis, rheumatoid arthritis, or ankylosing spondylitis (Beaudart et al., 2025).

LDH is classified within the TCM categories of “low back pain” and “lumbar paralysis.” TCM theory holds that most low back pain is related to kidney deficiency, arising from qi stagnation and blood stasis (Zhan et al., 2022). Jintiange (JTG) capsules are recognized for dispelling wind, relieving pain, and strengthening bones; their relatively rich collagen and calcium content contribute significantly to skeletal health^[19]^. Previous studies have shown that JTG exerts analgesic effects by regulating the GPL/PKC/TRPV1 pathway, significantly reducing levels of inflammatory mediators in CFA-induced mouse models and LPS-stimulated RAW 264.7 cells (Shen et al., 2026). In addition, Liu et al. confirmed through a systematic literature review and meta-analysis that JTG significantly improves bone mineral density in the femoral neck and lumbar spine, thereby alleviating pain (Li et al., 2025). Additionally, in a randomized controlled trial, Shu et al. found that JTG capsules improved overall clinical efficacy for osteoporotic vertebral compression fractures and offered advantages in enhancing imaging and physiological indicators, including anterior vertebral height, Cobb’s angle, and bone density (Shu and Zhang, 2022). Preliminary clinical studies further indicate that JTG capsules improve function and alleviate pain in LDH patients while reducing levels of inflammatory mediators like PGE2, NPY, and 5-HT. Basic research confirms that JTG capsules elevate blood calcium and bone density, promote bone formation, and inhibit bone resorption by modifying trabecular bone structure and enhancing enzyme activity^[21]^, thereby validating the correspondence between the capsule’s kidney-tonifying and bone-strengthening effects and the “kidney deficiency” syndrome in LDH. In a separate 4-week double-blind controlled trial involving 54 patients, Fu et al.explored celecoxib as an adjuvant for axial undifferentiated spondyloarthritis (axuSpA), demonstrating that the combination of Qiangji Recipe (QR) and celecoxib capsules was more effective than celecoxib alone (He and Fan, 2015). Moreover, a systematic review and meta-analysis by Zeng et al. indicated that combining sodium hyaluronate with celecoxib capsules was superior to control in improving the Lysholm knee score and clinical efficacy, while reducing VAS scores and adverse reactions in knee osteoarthritis treatment (Zeng et al., 2024). Our study shows that, for both primary and secondary outcomes, the TG exhibited significantly decreased VAS and numbness scores (*P <* 0.05) and significantly increased JOA, MCS, and PCS scores (*P <* 0.05) after 8 weeks compared to baseline. These results confirm the advantages of JTG capsules in alleviating pain, enhancing lumbar mobility. Furthermore, the TG consistently achieved higher scores across outcomes at 2 weeks, 8 weeks, and 2 weeks post-discontinuation compared to the CG. This difference may be attributed to several factors: the drug provides easily absorbable organic calcium and bone-essential trace elements; JTG capsules effectively alleviate symptoms like low back pain and numbness; and the medication is convenient, well-tolerated, and associated with few adverse reactions, thereby improving patient adherence (Huang et al., 2019; Arabmotlagh et al., 2019).

Lumbar spine stability depends on the paraspinal musculature, while lumbar range of motion (ROM) provides a complementary measure of functional status. After correction of the previous transcription error and application of baseline-adjusted multiple imputation combined with analysis of covariance (MI/ANCOVA), both bilateral lumbar muscle elastic-modulus measures and all four ROM outcomes at week 8 favored the JTG-containing regimen after Holm correction in the present analysis. These objective secondary outcomes should nevertheless be interpreted cautiously because they remain secondary endpoints and were assessed in an open-label trial without blinded outcome assessment.

The numbness outcome showed a distinct temporal pattern. Between-group differences favored the treatment group at weeks 2 and 8, whereas the difference was no longer statistically significant at the 2-week post-treatment follow-up after Bonferroni correction. Because the numbness score was an ordinal variable and subsequent observations were unavailable for the seven participants who discontinued follow-up, a non-parametric available-case approach was used rather than treating the score as a normally distributed continuous variable.

This pattern may suggest that sensory symptoms recover more slowly than pain; however, mechanistic interpretations should remain cautious because the study was not designed to distinguish inflammatory pain from mechanisms related to recovery from nerve injury. Accordingly, the absence of a statistically significant between-group difference in numbness after treatment discontinuation is better interpreted as an outcome-specific temporal observation rather than as evidence supporting a particular biological mechanism.

These findings also indicate that persistence of a treatment effect should not be inferred solely from statistically significant within-group changes. Although both groups showed changes over time in the complete-case analysis, the clinically more relevant comparison is the between-group contrast, which was not statistically significant for numbness at follow-up.

Accordingly, the numbness findings were retained as secondary or exploratory evidence and were not used to strengthen the primary JOA efficacy claim. Interpretation of the primary findings remained based on the baseline-adjusted between-group treatment effect for JOA and the corresponding sensitivity analyses addressing missing data.

Differences between subjective and objective outcomes do not exclude the possibility of expectation, performance, or detection bias. In particular, the open-label design and unequal pill burden between groups may have influenced patient-reported outcomes, and outcome assessors were not blinded. Therefore, even though some objective secondary outcomes showed between-group differences, these findings cannot be taken as evidence that expectation bias or other biases associated with the open-label design were absent.

Potential drug-drug interactions should also be considered when interpreting the present add-on design. Celecoxib is primarily metabolized through CYP2C9 and is known to inhibit CYP2D6, whereas eperisone undergoes extensive first-pass biotransformation involving several CYP enzymes, particularly CYP2J2 and CYP3A4. In contrast, JTG is a complex pharmaceutical preparation containing mineral components and protein/peptide constituents,and human pharmacokinetic data sufficient to characterize its effects on CYP enzymes or drug transporters are not currently available. Thus, there is no direct evidence demonstrating a clinically important pharmacokinetic interaction between JTG and either celecoxib or eperisone, but such an interaction cannot be formally excluded. Existing studies have reported clinical coadministration of celecoxib and eperisone (Gong et al., 2013), and pharmacokinetic studies of eperisone with other NSAIDs, such as aceclofenac and pelubiprofen (Kim et al., 2013; Ryu et al., 2017), have not shown clinically significant pharmacokinetic interactions; these findings are reassuring but indirect and cannot be extrapolated to JTG. From a pharmacodynamic perspective, the analgesic and anti-inflammatory activity of JTG may be additive to that of celecoxib, while its effects on musculoskeletal symptoms may complement those of eperisone. Such additive or synergistic effects may contribute to the clinical benefit observed in the treatment group. Therefore, the present results should be interpreted as evidence for the tested combination regimen rather than as proof of an independent JTG effect.

This study has several important limitations. First, the single-center design and relatively small sample size may limit the generalizability of the findings, and larger multicenter studies are warranted. Second, the study used an open-label add-on design without a matching placebo. The treatment group received an additional nine JTG capsules daily, resulting in unequal pill burden between groups and introducing the possibility of expectation and performance bias. In addition, outcome assessors were not blinded, and detection bias therefore cannot be excluded. Although between-group differences were also observed in several objectively measured secondary outcomes, these findings do not eliminate the potential biases associated with the open-label design. Future double-blind, double-dummy, placebo-controlled trials are needed to confirm the findings.

Third, attrition was unequal between groups, with two participants withdrawing from the treatment group and five from the control group. Although all randomized participants were retained in the ITT population and the mixed-effects models incorporated all available longitudinal observations under a missing-at-random assumption, informative dropout cannot be excluded. Complete-case analyses were therefore performed as sensitivity analyses, and the results should be interpreted in light of the unequal attrition pattern.

Fourth, multiple secondary outcomes were evaluated, which increases the possibility of chance findings despite multiplicity adjustment. Although Holm correction was applied to the week 8 secondary-outcome family, these secondary findings should still be interpreted cautiously.

Fifth, JTG was administered in addition to eperisone and short-term celecoxib rather than as monotherapy. Therefore, the observed between-group differences reflect the effect of the tested combination regimen and cannot be attributed solely to an independent effect of JTG.

Sixth, this trial was not designed as a dedicated pharmacokinetic or drug–drug interaction study. No serial plasma concentration measurements of celecoxib, eperisone, or identifiable JTG constituents were obtained. Consequently, pharmacokinetic interactions cannot be formally excluded, nor can the independent pharmacodynamic effect of JTG be distinguished from additive or synergistic effects arising from the combination regimen. Dedicated pharmacokinetic and mechanistic interaction studies are warranted.

## 5 Conclusion

In the intention-to-treat analysis, the JTG-containing regimen was associated with greater improvement in the primary JOA outcome and several secondary outcomes compared with conventional therapy alone. Complete-case sensitivity analyses produced treatment-effect estimates that were broadly consistent with the primary ITT findings. However, because of the open-label, non-placebo-controlled add-on design, lack of assessor blinding, differential attrition, multiplicity, and the relatively small single-center sample, these findings should be interpreted cautiously. Larger multicenter, double-blind, placebo-controlled trials are required to confirm the observed treatment effects.

## Data Availability

The original contributions presented in the study are included in the article/Supplementary Material, further inquiries can be directed to the corresponding author.
