# Supplementary material for: Jintiange capsule as add-on therapy for lumbar disc herniation: an open-label randomized controlled trial
Source: Front Pharmacol. 2026 Oct 8;17:1837527. doi: 10.3389/fphar.2026.1837527 (PMC13687347; doi:10.3389/fphar.2026.1837527)
Supplement: Supplementary file 1 [file Supplementaryfile1.pdf]

安徽中医药大学第一附属医院医学伦理委员会

伦理审查意见同意函

伦理审查同意号（原伦理审查批件号）	2022AH-49
主要研究者	张建华
项目名称	金天格胶囊用于腰椎间盘突出症的随机平行对照临床研究
资助来源	金花企业（集团）股份有限公司
研究单位	安徽中医药大学第一附属医院
会议审查时间	2022 年 6 月 23 日
会议地点	合肥梅山路 117 号安徽中医药大学第一附属医院
审查类别/审查方式	初审审查/会议审查　复审/快速审查
到会情况	伦理委员会委员共 13 人，到会 12 人，缺席 1 人，投票 12 人
票数	作必要的修改后同意 12 票
审查/同意文件	1. 伦理审查申请表<br>2. 临床研究方案（版本号/日期：V2.0/2022 年 6 月 30 日）<br>3. 知情同意书（版本号/日期：V2.0/2022 年 6 月 30 日）<br>4. 病例报告表<br>5. 主要研究者专业履历
审查意见	根据国家药监局，国家卫生健康委发布的“药物临床试验质量管理规范”(2020 年)，“药物临床试验伦理审查工作指导原则”(2010 年)，原卫计委“涉及人的生物医学研究伦理审查办法”(2016 年)，国家中医药管理局“中医药临床研究伦理审查管理规范”(2010 年)，以及世界医学会《赫尔辛基宣言》(2008) 等，经本伦理委员会审查，按所同意的临床研究方案、知情同意书开展本项研究。

伦理委员会声明	请遵循 GCP 原则、遵循伦理委员会同意的方案开展临床研究，保护受试者的健康与权利。<br>研究开始前，请申请人尽可能完成临床试验注册。<br>研究过程中若变更主要研究者，对临床研究方案、知情同意书、招募材料等的任何修改，请申请人提交修正案审查申请。<br>请按照伦理委员会规定的年度/定期跟踪审查频率，审查人在截止日期前 1 个月提交研究进展报告；申办者应当向组长单位伦理委员会提交各中心研究进展的汇总报告；当出现任何可能显著影响试验进行、或增加受试者危险的情况时，请申请人及时向伦理会员会提交书面报告。<br>研究纳入了不符合纳入标准或符合排除标准的受试者，符合中止试验规定而未让受试者退出研究，给予错误治疗或剂量，给予方案禁止的合并用药等没有遵从方案开展研究的情况；或可能对受试者的权益/健康以及研究的科学性造成不良影响等违背 GCP 原则的情况，请申办者/监察员/研究者提交违背方案报告。<br>申请人暂停或提前终止临床研究，请及时提交暂停/终止研究报告。完成临床研究，请提交结题报告。

有效期	有效期 2 年（两年内有效，2022 年 7 月 4 日至 2024 年 7 月 4 日）	年度/定期跟踪审查频率	12 个月
伦理委员会联系人及联系电话		徐桂琴：0551-62838532	
伦理委员会主任委员签字及日期		2022 年 7 月 4 日	
安徽中医药大学第一附属医院（安徽省中医院）医学伦理委员会（盖章）			
