# Supplementary material for: Jintiange capsule as add-on therapy for lumbar disc herniation: an open-label randomized controlled trial
Source: Front Pharmacol. 2026 Oct 8;17:1837527. doi: 10.3389/fphar.2026.1837527 (PMC13687347; doi:10.3389/fphar.2026.1837527)
Supplement: Supplementary file 2 [file Supplementaryfile2.pdf]

核准日期：2007年07月12日
修改日期：2024年05月29日

金花®

用微信扫描二维码
可查看电子说明书

金天格胶囊说明书

请仔细阅读说明书并在医师指导下使用

警示语

【药品名称】通用名称：金天格胶囊
汉语拼音：Jintiange Jiaonang

【成　　份】人工虎骨粉。

【性　　状】本品为硬胶囊，内容物为类白色或淡黄色粉末；气微，无味。

【功能主治】具有健骨作用，用于腰背疼痛，腰膝酸软，下肢痿弱，步履艰难等症状的改善。

【规　　格】每粒装0.4g(含氨基酸65mg，钙55mg，磷35mg)

【用法用量】口服。一次3粒，一日3次。一个疗程为3个月。

【不良反应】患者服药后可能会出现口干、恶心、呕吐等胃肠道不适，也可能会出现皮肤局部皮疹、瘙痒等皮肤系统的反应。

【禁　　忌】对本品过敏者禁用。

【注意事项】1.因服药后可能会引起口干，建议服用本品期间多饮水；
2.过敏体质者慎服，或在医师、药师的指导下使用；
3.本品性状发生改变时禁止使用；
4.请将本品放在儿童不能接触的地方。

【临床试验】本品于1997年经中华人民共和国卫生部药政管理局批准进行过660例临床试验。

【药理毒理】临床前药效学试验表明，本品可使卡拉胶致大鼠足趾肿胀及巴豆油致小鼠耳肿胀的肿胀度减轻，使大鼠肉芽抑制；在物理法及化学法致痛试验中，本品可使小鼠痛阈值提高，使腹腔注射醋酸和酒石酸锑钾所致的小鼠扭体次数减少；在小鼠自主活动试验及与戊巴比妥钠的协同作用试验中，本品可使小鼠的自发活动减少，与戊巴比妥钠具有协同作用；在维甲酸和糖皮质激素致大鼠骨质疏松模型上，本品具有一定促进骨生长的作用。

【贮　　藏】密封，置阴凉干燥处。

【包　　装】铝塑板。3×12粒/板/盒。

【有 效 期】24个月

【执行标准】国家药品监督管理局国家药品标准
YBZ01592003-2009Z-2019

【批准文号】国药准字Z20030080

【药品上市许可持有人】
企业名称：金花企业（集团）股份有限公司西安金花制药厂
注册地址：西安市高新区高新技术产业开发区科技四路202号

【生产企业】
企业名称：金花企业（集团）股份有限公司西安金花制药厂
生产地址：西安市高新技术产业开发区科技四路202号
邮政编码：710065　　电话号码：029-82200221
传真号码：029-81778690　　网址：www.ginwa.com.cn
客户服务热线：4008870015

金花　GINWA　金花企业(集团)股份有限公司　西安金花制药厂

夸克扫描王
极速扫描，就是高效
