# Supplementary material for: Jintiange capsule as add-on therapy for lumbar disc herniation: an open-label randomized controlled trial
Source: Front Pharmacol. 2026 Oct 8;17:1837527. doi: 10.3389/fphar.2026.1837527 (PMC13687347; doi:10.3389/fphar.2026.1837527)
Supplement: Supplementary file 3 [file Supplementaryfile3.pdf]

Table 1. Normality test of JOA scores

Tests of Normality

	group	Kolmogorov-Smirnov[a]			Shapiro-Wilk		
		Statistic	df	Sig.	Statistic	df	Sig.
Baseline	treatment	.089	30	.200*	.975	30	.691
	control	.178	27	.028	.932	27	.076
JOA2week	treatment	.125	30	.200*	.956	30	.244
	control	.125	27	.200*	.970	27	.594
JOA8week	treatment	.152	30	.076	.931	30	.053
	control	.123	27	.200*	.953	27	.253
JOA10week	treatment	.128	30	.200*	.938	30	.078
	control	.097	27	.200*	.966	27	.493

*. This is a lower bound of the true significance.

a. Lilliefors Significance Correction

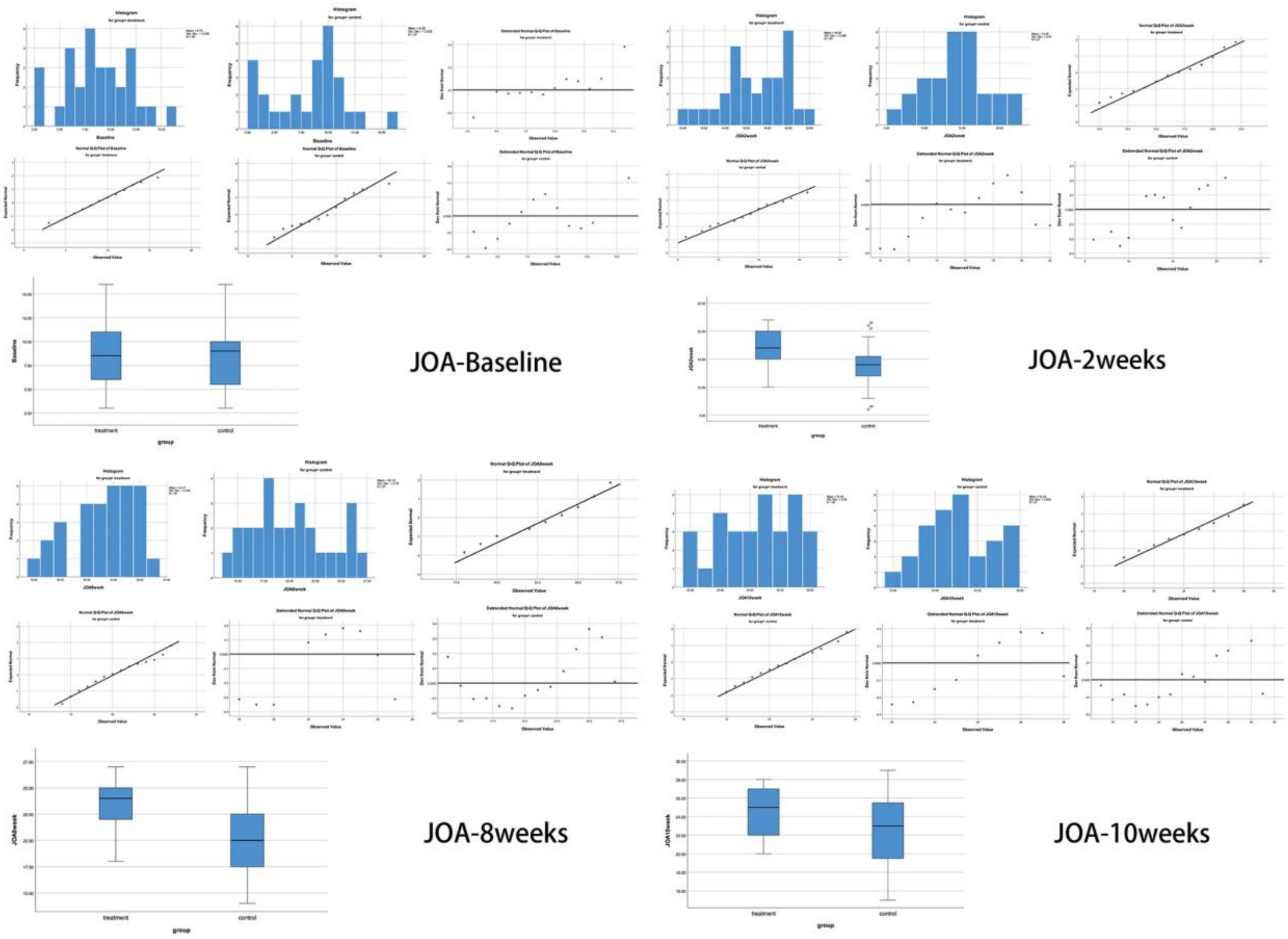

Figure 1. Normality distribution of JOA scores

Table 2. Normality test for age

Tests of Normality

	group	Kolmogorov-Smirnov[a]			Shapiro-Wilk		
		Statistic	df	Sig.	Statistic	df	Sig.
Age	treatment	.121	30	.200*	.956	30	.251
	control	.098	27	.200*	.969	27	.566

*. This is a lower bound of the true significance.

a. Lilliefors Significance Correction

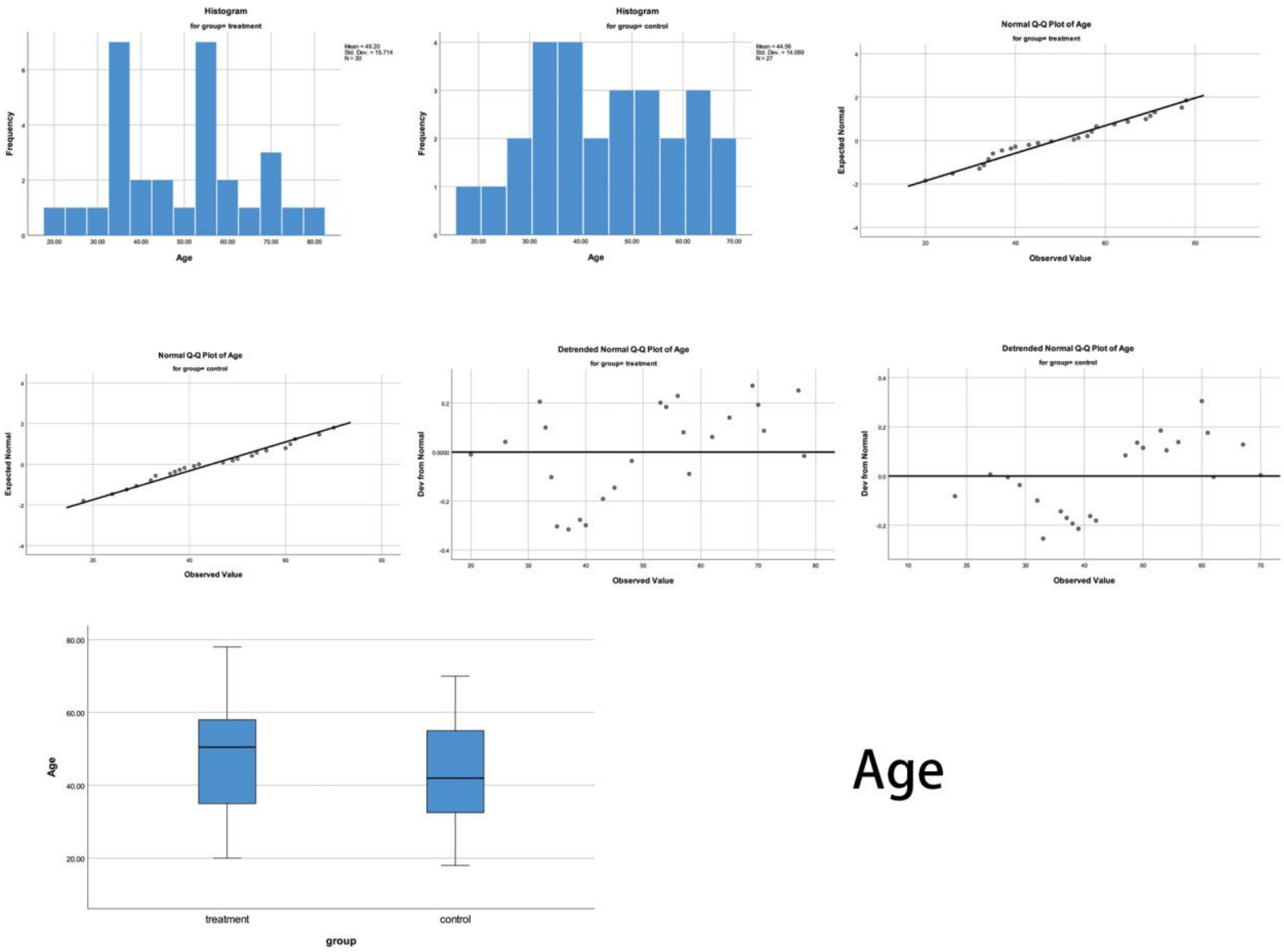

Figure 2. Normality assessment for age

Table 3. Normality test for disease course

Tests of Normality

		Kolmogorov-Smirnov[a]			Shapiro-Wilk		
	group	Statistic	df	Sig.	Statistic	df	Sig.
course	treatment	.315	30	.000	.804	30	.000
	control	.232	27	.001	.775	27	.000

a. Lilliefors Significance Correction

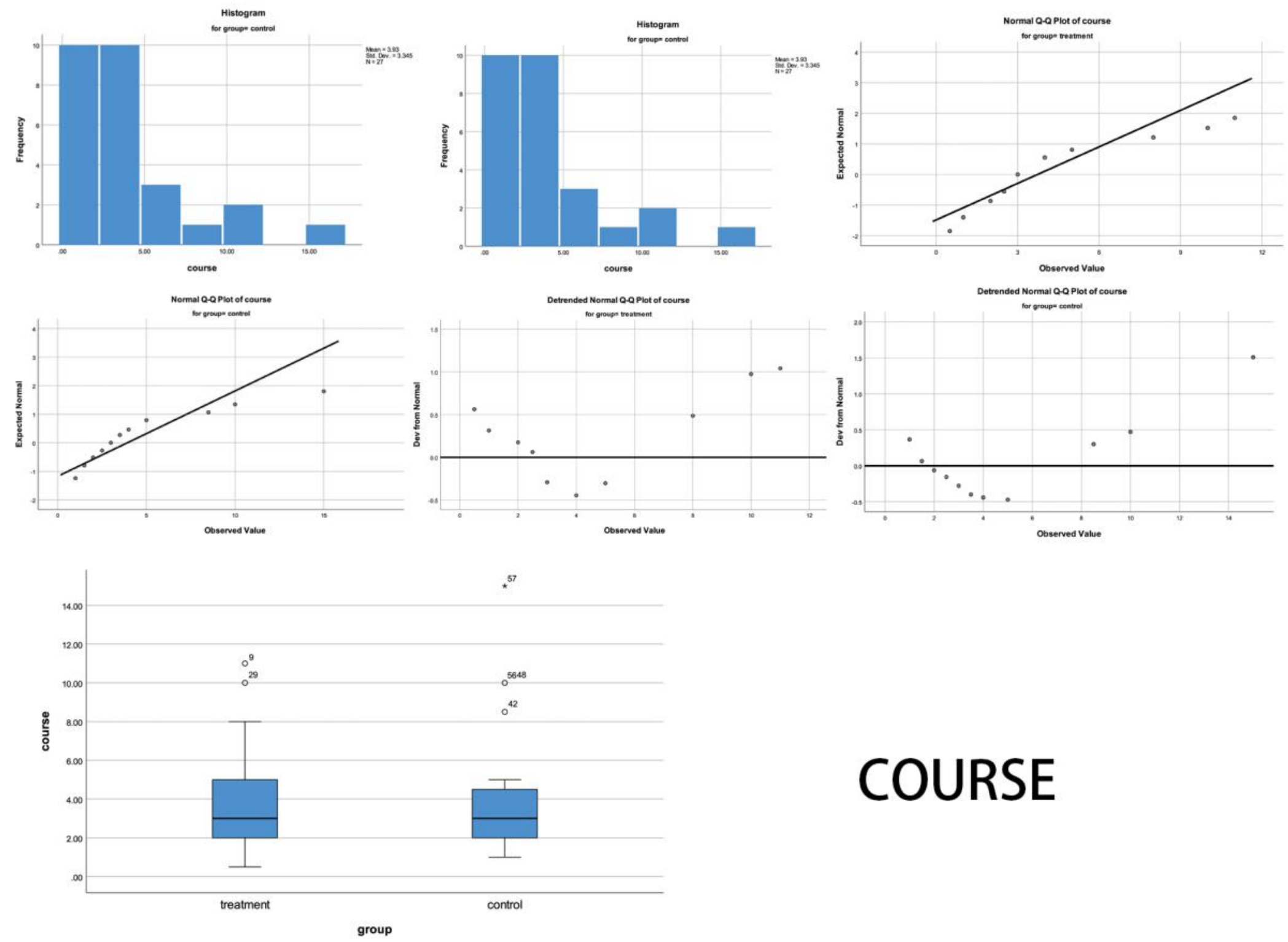

Figure 3. Normality assessment for disease course

Table 4. Normality test for VAS scores

Tests of Normality

	group	Kolmogorov-Smirnov[a]			Shapiro-Wilk		
		Statistic	df	Sig.	Statistic	df	Sig.
baseline	treatment	.136	30	.167	.946	30	.131
	control	.180	27	.025	.931	27	.074
VAS2weeks	treatment	.148	30	.093	.965	30	.424
	control	.139	27	.194	.942	27	.137
VAS8weeks	treatment	.139	30	.142	.958	30	.271
	control	.182	27	.022	.929	27	.064
VAS10weeks	treatment	.171	30	.025	.950	30	.167
	control	.140	27	.186	.945	27	.160

a. Lilliefors Significance Correction

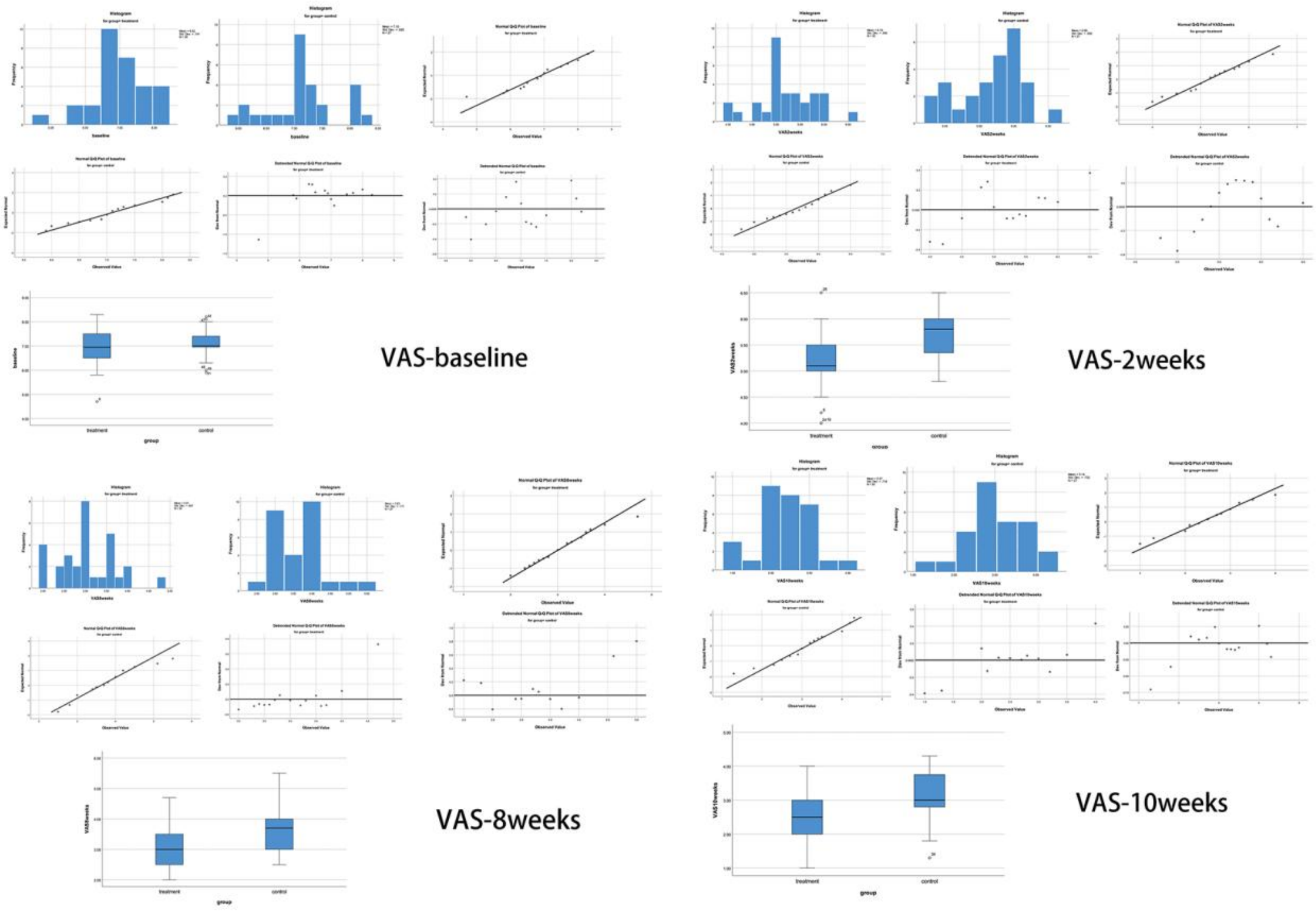

Figure 4. Normality assessment for VAS scores

Table 5. Normality test for muscle elastic modulus

Tests of Normality

		Kolmogorov-Smirnov[a]			Shapiro-Wilk		
	Group	Statistic	df	Sig.	Statistic	df	Sig.
Leftbaseline	treatment	.115	30	.200*	.960	30	.317
	control	.137	27	.200*	.927	27	.060
Rightbaseline	treatment	.102	30	.200*	.977	30	.733
	control	.110	27	.200*	.963	27	.438
Left8weeks	treatment	.115	30	.200*	.936	30	.073
	control	.088	27	.200*	.978	27	.806
Right8weeks	treatment	.086	30	.200*	.970	30	.537
	control	.118	27	.200*	.951	27	.224

*. This is a lower bound of the true significance.

a. Lilliefors Significance Correction

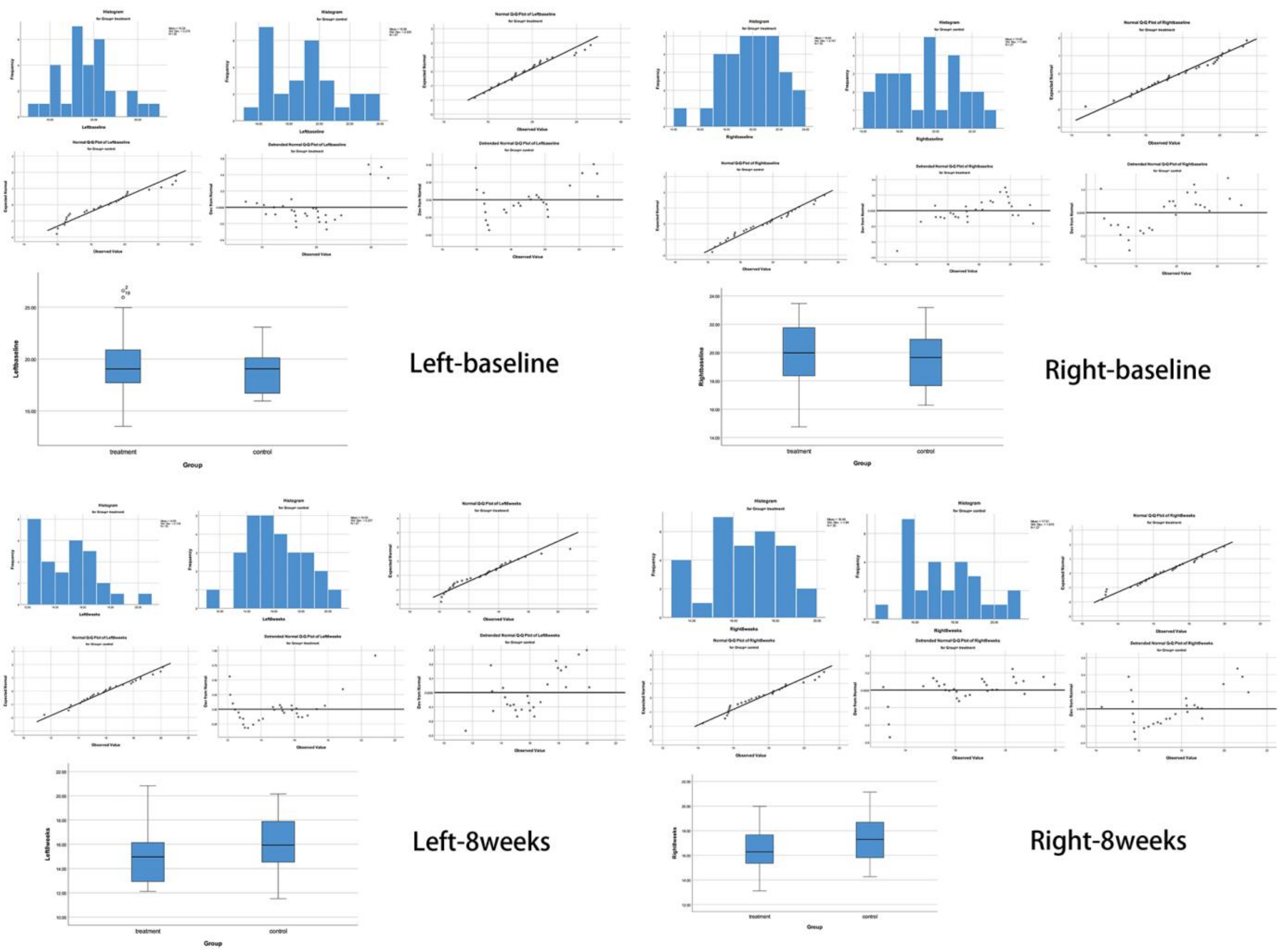

Figure 5. Normality assessment for muscle elastic modulus

Table 6. Normality test for lumbar range of motion

Tests of Normality

		Kolmogorov-Smirnov[a]			Shapiro-Wilk		
	Group	Statistic	df	Sig.	Statistic	df	Sig.
Anteflexion	treatment	.107	30	.200*	.976	30	.724
	control	.132	27	.200*	.951	27	.229
Backextension	treatment	.118	30	.200*	.937	30	.077
	control	.102	27	.200*	.986	27	.961
Leftflexion	treatment	.100	30	.200*	.973	30	.610
	control	.127	27	.200*	.937	27	.105
Rightflexion	treatment	.101	30	.200*	.967	30	.454
	control	.110	27	.200*	.931	27	.072
Ante8weeks	treatment	.085	30	.200*	.972	30	.596
	control	.094	27	.200*	.956	27	.295
Back8weeks	treatment	.155	30	.065	.948	30	.146
	control	.073	27	.200*	.981	27	.881
Left8weeks	treatment	.134	30	.177	.936	30	.071
	control	.075	27	.200*	.965	27	.476
Right8weeks	treatment	.166	30	.034	.952	30	.188
	control	.189	27	.014	.937	27	.102

*. This is a lower bound of the true significance.

a. Lilliefors Significance Correction

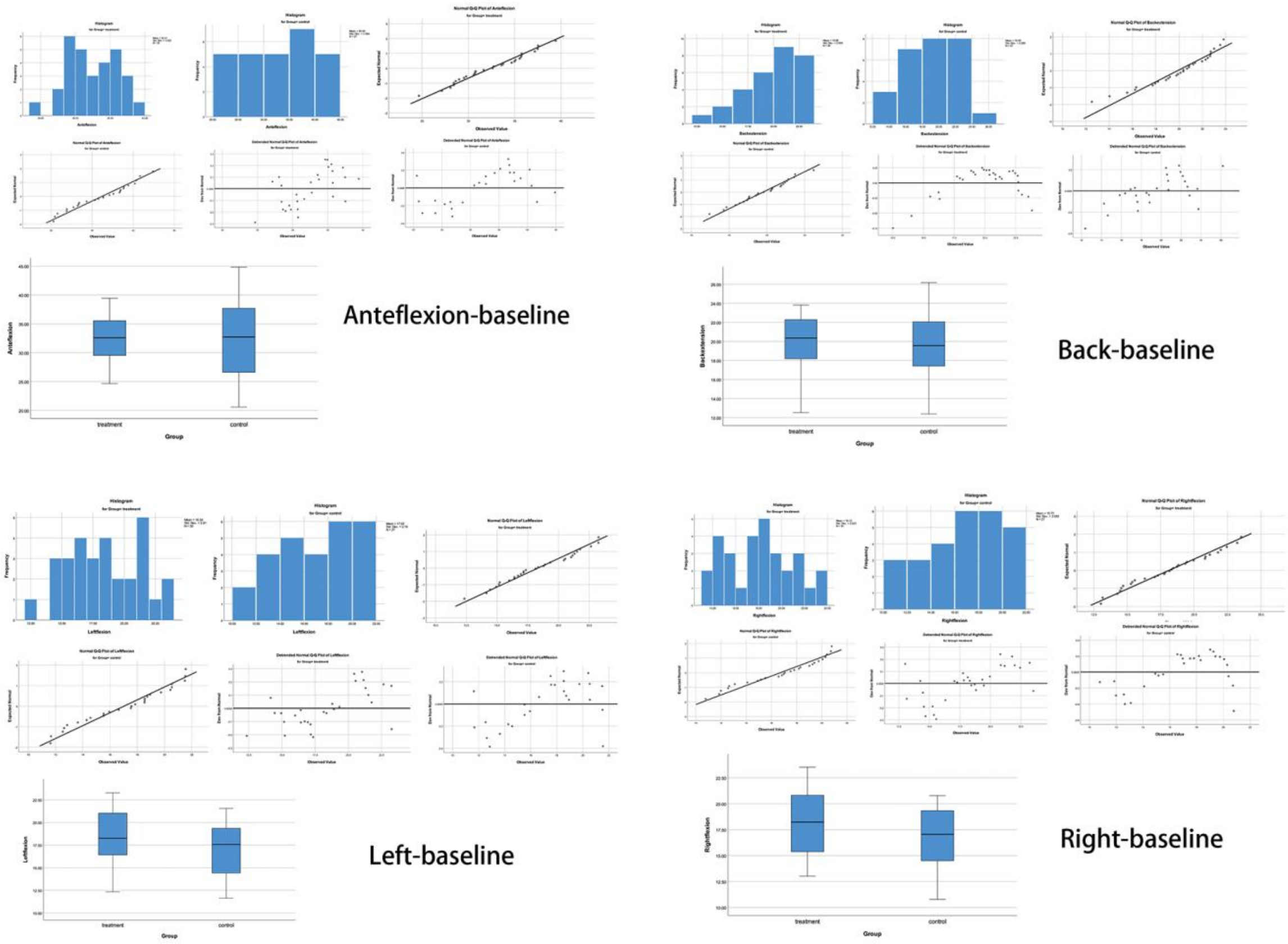

Figure 6. Normality assessment for baseline lumbar range of motion

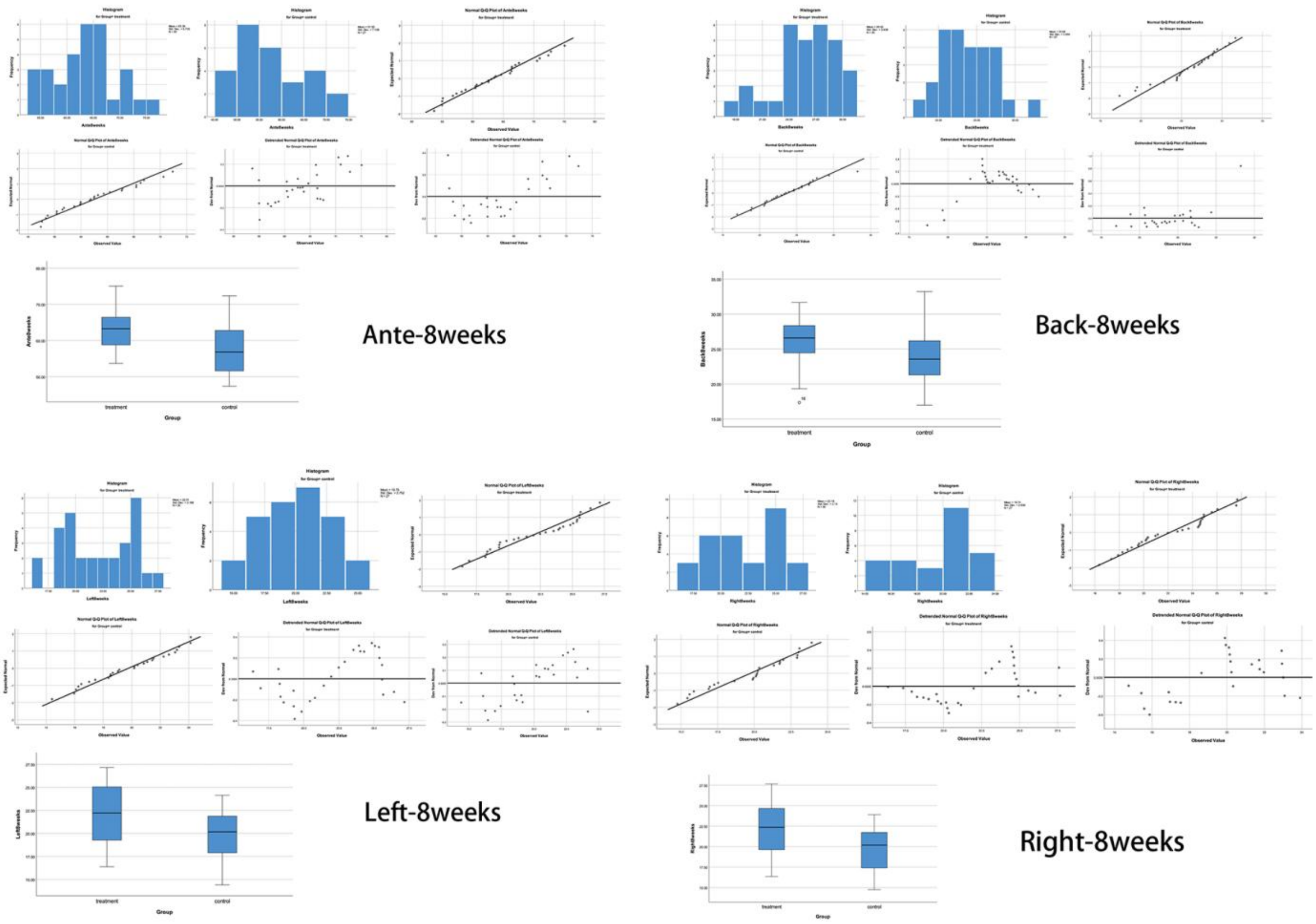

Figure 7. Normality assessment for lumbar range of motion at 8-week time-point

Table 7. Normality test for MCS scores

Tests of Normality

	Group	Kolmogorov-Smirnov[a]			Shapiro-Wilk		
		Statistic	df	Sig.	Statistic	df	Sig.
MCS	treatment	.125	30	.200*	.972	30	.584
	control	.107	27	.200*	.960	27	.364
MCS2weeks	treatment	.065	30	.200*	.982	30	.877
	control	.163	27	.065	.961	27	.388
MCS8weeks	treatment	.125	30	.200*	.942	30	.103
	control	.069	27	.200*	.983	27	.917
MCS10weeks	treatment	.081	30	.200*	.974	30	.652
	control	.089	27	.200*	.975	27	.734

*. This is a lower bound of the true significance.

a. Lilliefors Significance Correction

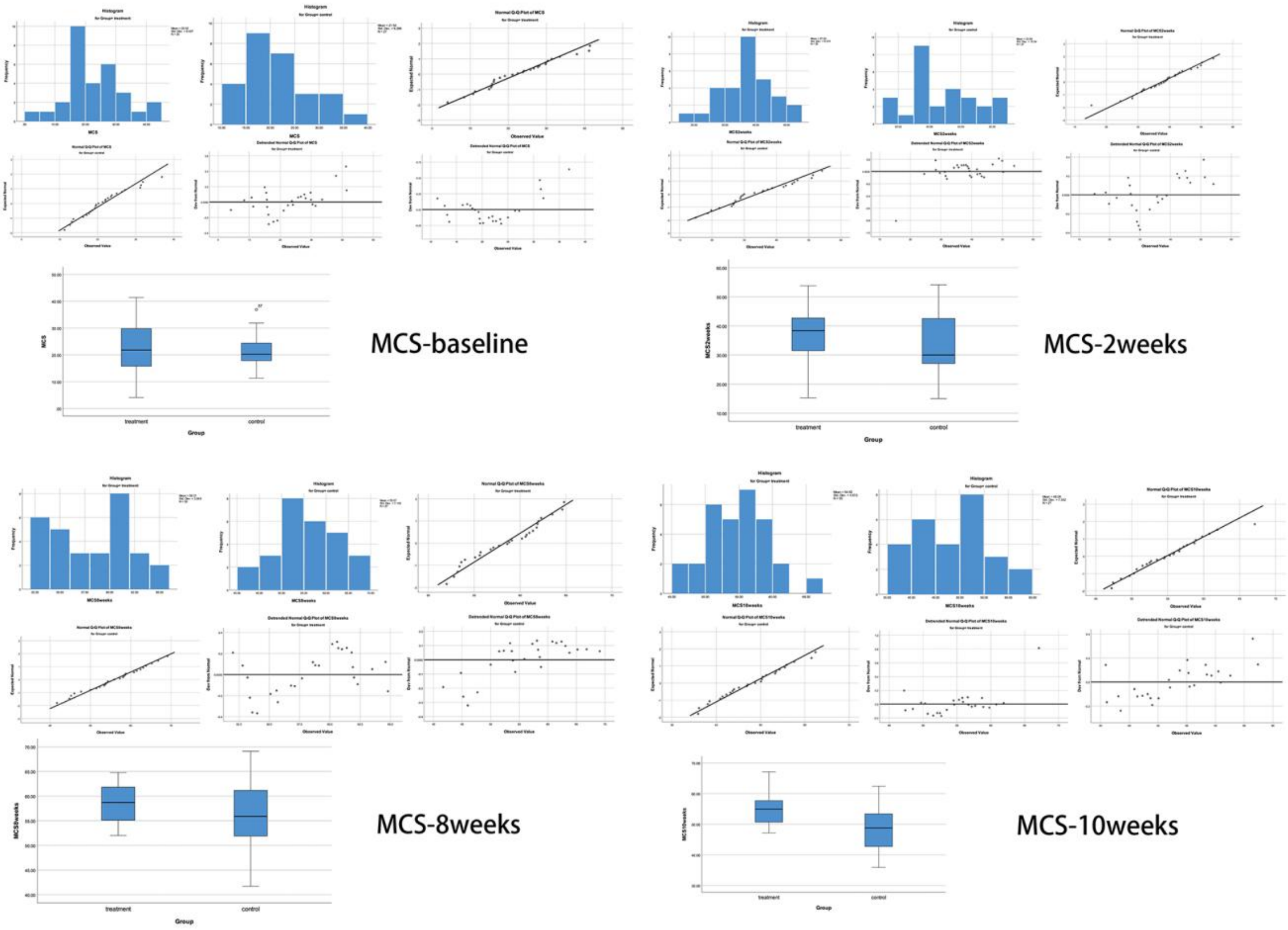

Figure 8. Normality assessment for MCS scores

Table 8. Normality test for PCS scores

Tests of Normality

		Kolmogorov-Smirnov[a]			Shapiro-Wilk		
	Group	Statistic	df	Sig.	Statistic	df	Sig.
PCS	treatment	.134	30	.175	.948	30	.150
	control	.126	27	.200*	.971	27	.618
PCS2weeks	treatment	.166	30	.034	.936	30	.072
	control	.150	27	.123	.932	27	.079
PCS8weeks	treatment	.082	30	.200*	.972	30	.610
	control	.110	27	.200*	.962	27	.416
PCS10weeks	treatment	.083	30	.200*	.985	30	.929
	control	.115	27	.200*	.979	27	.835

*. This is a lower bound of the true significance.

a. Lilliefors Significance Correction

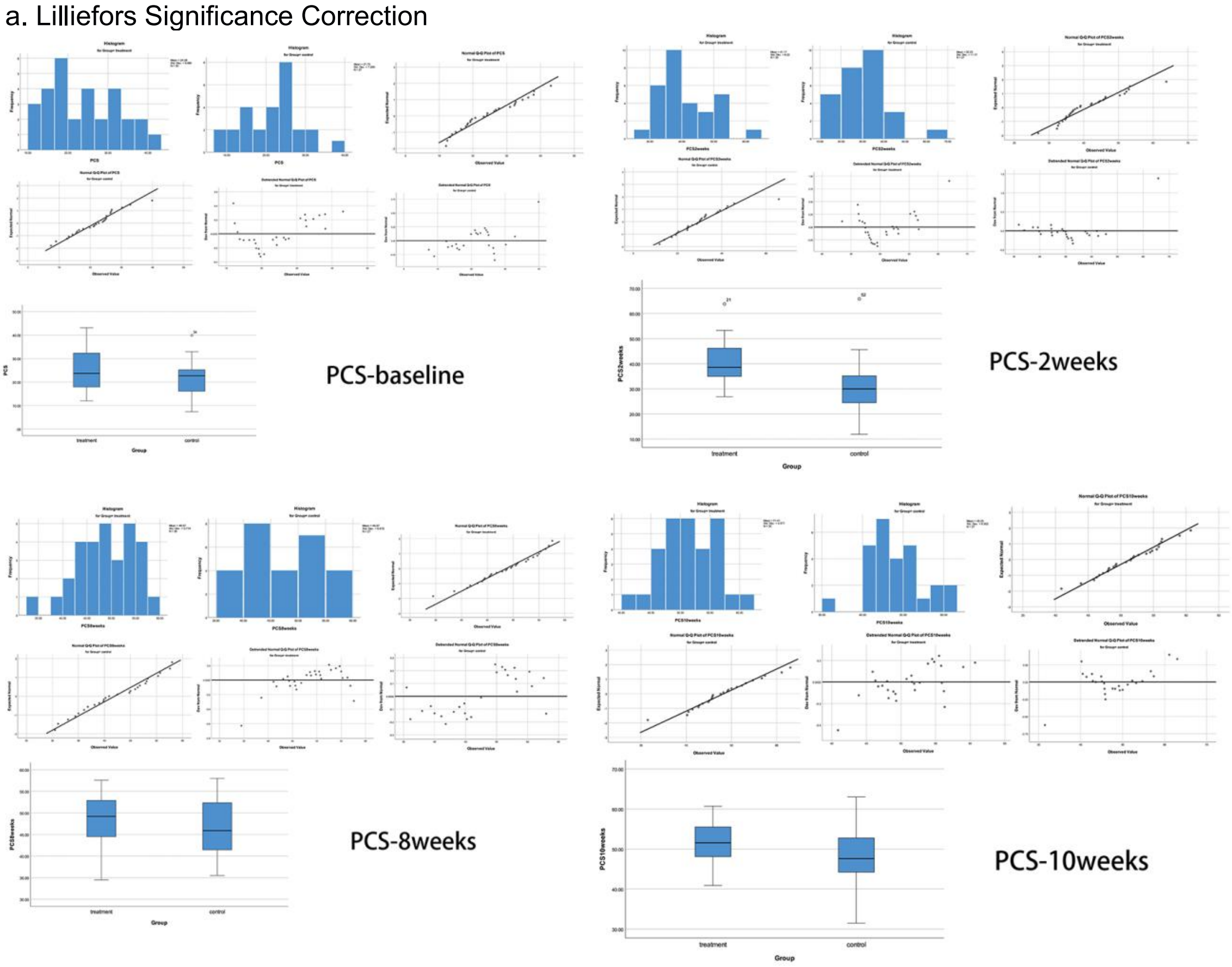

Figure 9. Normality assessment for PCS scores

Table 9. Normality test for Likert- scale numbness scores

Tests of Normality

		Kolmogorov-Smirnov[a]			Shapiro-Wilk		
	Group	Statistic	df	Sig.	Statistic	df	Sig.
likertbaseline	treatment	.372	30	.000	.632	30	.000
	control	.366	27	.000	.634	27	.000
likert2weeks	treatment	.322	30	.000	.772	30	.000
	control	.340	27	.000	.754	27	.000
likert8weeks	treatment	.354	30	.000	.637	30	.000
	control	.354	27	.000	.743	27	.000
likert10weeks	treatment	.287	30	.000	.798	30	.000
	control	.283	27	.000	.782	27	.000

a. Lilliefors Significance Correction

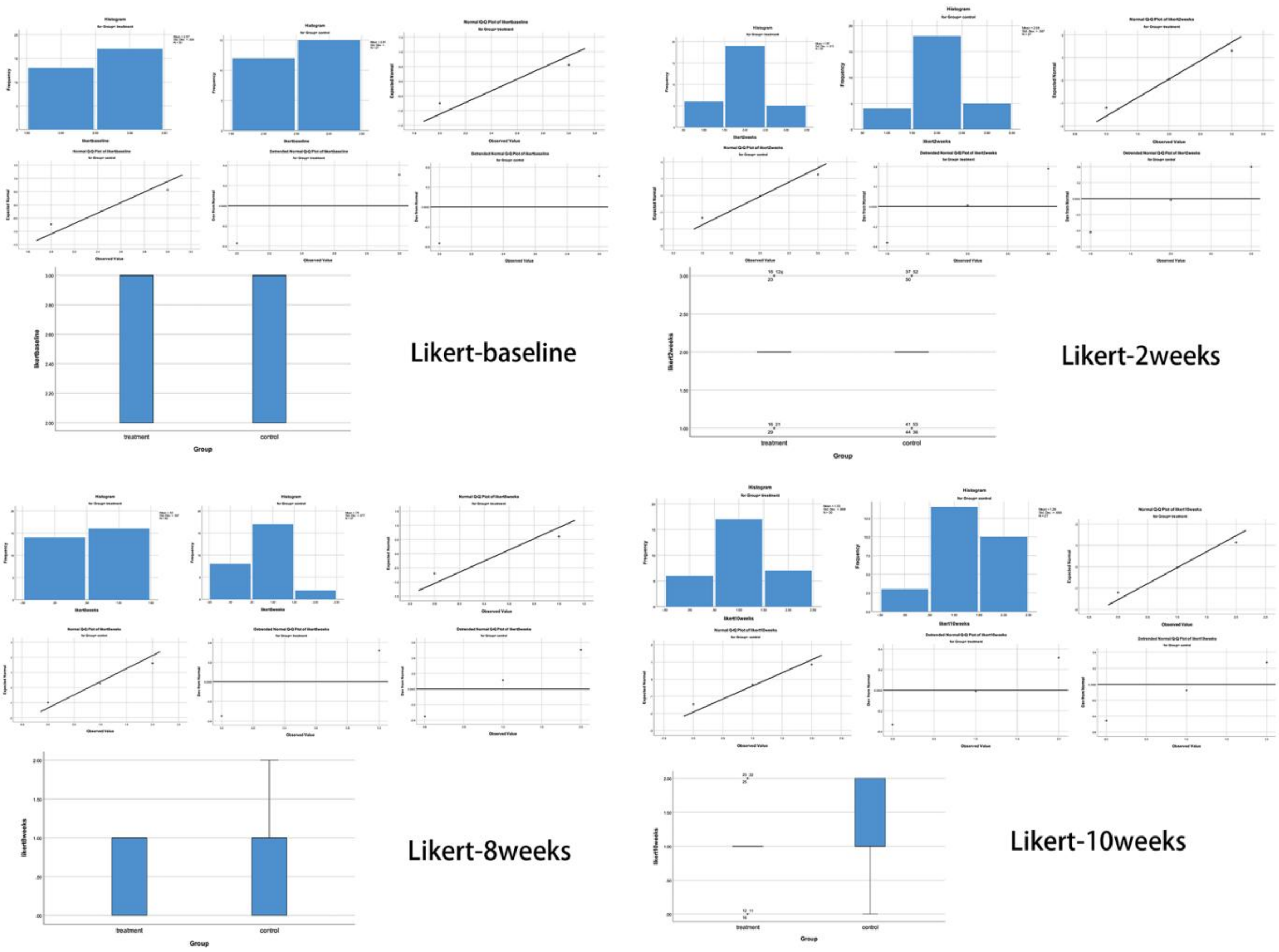

Figure 10. Normality assessment for Likert- scale numbness scores
