# Supplementary material for: Jintiange capsule as add-on therapy for lumbar disc herniation: an open-label randomized controlled trial
Source: Front Pharmacol. 2026 Oct 8;17:1837527. doi: 10.3389/fphar.2026.1837527 (PMC13687347; doi:10.3389/fphar.2026.1837527)
Supplement: Supplementary file 4 [file Supplementaryfile4.pdf]

金花企业（集团）股份有限公司西安金花制药厂

检验报告书

编号：PQR 03 QC021-005

送检日期	2026-02-08	报告日期	2026-02-26
品　　名	金天格胶囊	规　　格	每粒装 0.4g
批　　号	260203	检验编号	CJT-26-008
检 品 量	24板	检品来源	固体车间
检验依据	《国家食品药品监督管理局国家药品标准YBZ01592003-2009Z-2019》		
检验项目	标准规定	检验结果	结论
【性　状】	本品为硬胶囊，内容物为类白色或淡黄色粉末；气微，无味	本品为硬胶囊，内容物为淡黄色粉末；气微，无味	符合规定
【鉴　别】			
鉴别1	应呈正反应	呈正反应	符合规定
鉴别2	在［含量测定］总氨基酸项下的色谱图中，供试品色谱中应呈现与对照品色谱峰保留时间相对应的色谱峰	在［含量测定］总氨基酸项下的色谱图中，供试品色谱中呈现与对照品色谱峰保留时间相对应的色谱峰	符合规定
【检　查】			
水分	不得过8.0%	3.0%	符合规定
装量差异	±10%	未超出装量差异限度	符合规定
崩解时限	应在30分钟内全部崩解	16分钟	符合规定
【微生物限度】	需氧菌总数≤10^3cfu/g	50cfu/g	符合规定
	霉菌和酵母菌总数≤10^2cfu/g	40cfu/g	符合规定
	大肠埃希菌：不得检出/g	未检出/g	符合规定
	沙门菌：不得检出/10g	未检出/10g	符合规定
【特征图谱】	供试品特征图谱中应呈现与对照药材中的7个特征峰保留时间相对应的色谱峰；其中以对照特征图谱中的3号峰为S峰，计算各特征峰与S峰的相对保留时间，其相对保留时间应在规定值的±5%之内	供试品特征图谱中呈现与对照药材中的7个特征峰保留时间相对应的色谱峰；其中以对照特征图谱中的3号峰为S峰，计算各特征峰与S峰的相对保留时间，其相对保留时间均在规定值的±5%之内	符合规定
【含量测定】			
总钙	本品每粒含钙（Ca）不得少于55.0mg	76.5mg/粒	符合规定
总磷	本品每粒含磷（P）应为31.5～38.5mg	35.7mg/粒	符合规定
总氨基酸	本品每粒含各氨基酸的总量应不得少于65.0mg	76.3mg/粒	符合规定
明胶空心胶囊铬《中国药典》2025年版四部	不得过0.0002%		
生产厂家	安徽黄山（批号：2510065）	0.00001%	符合规定
结论	本品按《国家食品药品监督管理局国家药品标准YBZ01592003-2009Z-2019》检验，结果符合规定		

质量部经理：陈建平　　复核人：刘亚锐　　检验人：[illegible]
