# Supplementary material for: Jintiange capsule as add-on therapy for lumbar disc herniation: an open-label randomized controlled trial
Source: Front Pharmacol. 2026 Oct 8;17:1837527. doi: 10.3389/fphar.2026.1837527 (PMC13687347; doi:10.3389/fphar.2026.1837527)
Supplement: Supplementary file 5 [file Supplementaryfile5.pdf]

金天格胶囊主要动物源药用成分性质与来源说明

本企业生产的金天格胶囊，国药准字 Z20030080，处方中的药用成分为人工虎骨粉。人工虎骨粉为国家批准的天然虎骨仿生替代药用原料，本品不含天然虎骨，原料组分不来源于老虎及其组织，不属于虎源动物源性药材。人工虎骨粉系依据天然虎骨的理化、药效特征仿生研制而成，为本药品处方内法定药用成分，其质量标准载入国家药品标准。本品生产全过程、质量控制活动均严格遵照获批的国家药品标准及现行药品监管法规、GMP 要求执行。企业已建立完整的上游供应商管理体系，保留原料药采购合同、检验报告、出入库记录、供应链溯源等全套质量文件。上述资料可供药品监管部门依法核查。供应商具体商业信息属于企业商业秘密，按法律法规规定，不对社会公众披露。

金花企业（集团）股份有限公司西安金花制药厂

日期：2026 年 9 月 10 日
